# The National Ecological Observatory Network’s soil metagenomes: assembly and basic analysis

**DOI:** 10.12688/f1000research.51494.1

**Published:** 2021-04-19

**Authors:** Zoey R. Werbin, Briana Hackos, Michael C. Dietze, Jennifer M. Bhatnagar

**Affiliations:** 1 Department of Biology, Boston University, Boston, MA, 02215, USA; 2 Department of Mathematics, University of Colorado, Boulder, Boulder, CO, 80309, USA; 3 Department of Earth & Environment, Boston University, Boston, MA, 02215, USA

**Keywords:** metagenomics, microbial ecology, soil microbiome, tutorial, workflow

## Abstract

The National Ecological Observatory Network (NEON) annually performs shotgun metagenomic sequencing to sample genes within soils at 47 sites across the United States. NEON serves as a valuable educational resource, thanks to its open data policies and programming tutorials, but there is currently no introductory tutorial for performing analyses with the soil shotgun metagenomic dataset. Here, we describe a workflow for processing raw soil metagenome sequencing reads using the Sunbeam bioinformatics pipeline. The workflow includes cleaning and processing raw reads, taxonomic classification, assembly into contigs, annotation of predicted genes using custom protein databases, and exporting assemblies to the KBase platform for downstream analysis. This workflow is designed to be robust to annual data releases from NEON, and the underlying Snakemake framework can manage complex software dependencies. The workflow presented here aims to increase the accessibility of NEON’s shotgun metagenome data, which can provide important clues about soil microbial communities and their ecological roles.

## Introduction

The soil microbiome is responsible for key ecological processes, such as decomposition and nitrogen cycling ( Allison *et al.* 2013). One powerful tool for studying the soil microbiome is shotgun metagenomic sequencing, in which all of the genetic material within the DNA extract of a soil sample is sequenced at once, without targeting specific organisms ( Quince *et al.* 2017, Pérez-Cobas *et al* 2020). The largest publicly available sequencing dataset of this type is updated annually by the National Ecological Observatory Network (NEON), which monitors ecological conditions at 47 terrestrial sites spanning 20 ecoclimatic domains across the US and its territories ( Keller *et al.* 2018). NEON is funded by the National Science Foundation (NSF), and collects soil samples and releases shotgun metagenomics data annually.

To date, the NEON soil metagenomics data can only be accessed in two formats: as completely raw reads released by NEON, or as processed files through the default protocols of the MG-RAST storage server. Neither format is suitable for most metagenomic analyses, which generally answer scientific questions using custom data processing pipelines that use specific algorithms and targeted reference databases ( Ladoukakis *et al.* 2014; Quince *et al.* 2017). To facilitate future scientific analysis, we present a workflow for taking raw sequences and generating a processed dataset that can be linked to other NEON data products, which include soil biogeochemistry, root measurements, or aboveground plant communities.

NEON data is a valuable resource for ecology and bioinformatics, thanks to its open access software, robust documentation, and educational resources ( Jones 2020). The pipeline that we present here is designed to complement existing NEON educational resources, such that users without prior bioinformatics experience may use this dataset to learn about microbial communities within the soil. We present code and explanations for each analysis step, including basic quality control (QC), assembling reads into larger genome fragments (“contig” assembly), predicting genes, quantifying gene counts for specific ecological or biogeochemical functions, and exporting to the KBase platform ( Arkin *et al.* 2018). We recommend the review by Pérez-Cobas *et al.* (2020) for an overview of software alternatives for each step of this shotgun metagenomics analysis.

## Methods

### Dataset description

Soil samples are collected annually from 47 NEON sites during peak greenness. Three samples are collected within a NEON plot at a sampling time point. Soil samples are collected up to 30cm below the soil surface, the organic (O) and the mineral (M) horizons (when present) are separated, and subsamples from each horizon are homogenized into one composite sample per horizon. Sample file names include the 4-letter site identifiers, horizons (O or M), and sampling date. Samples are frozen on dry ice until DNA extraction and preparation using the KAPA Hyper Plus kit (Kapa Biosystems). Samples from multiple sites are pooled into sets of 40 or 60 for sequencing, which is conducted on an Illumina NextSeq at the Battelle Memorial Institute (NEON Metagenomics Standard Operating Procedure, v.3). Since there is currently no versioned release of NEON’s metagenomic data, the pipeline described here is designed to be robust to processing new data as it is released from NEON, approximately annually (TOS Science Design for Terrestrial Microbial Diversity, NEON.DOC.000908).

### Operation

We assume a Linux operating system and command-line interface. Storage and RAM requirements will depend on the specific analyses performed and the number of samples analyzed. If using shared computing clusters, refer to the Sunbeam manual for cluster-specific options, which are necessary to take full advantage of multi-core processing.

### Implementation

Once sequences are downloaded, we use the software Sunbeam ( Clarke *et al.* 2019) to create a bioinformatic pipeline. Sunbeam links a variety of popular bioinformatics tools (e.g. BLAST, MegaHIT, Kraken2, Prodigal), and users can develop and share customized extensions for various purposes. Sunbeam, and its underlying Snakemake framework ( Köster *et al.* 2012), are designed to address common problems with software versioning and updating, as well as efficient data re-analysis (i.e. running the minimal tasks necessary to generate updated output files). In addition to Sunbeam’s default steps for cleaning and processing the raw reads, the pipeline below performs taxonomic classification or protein annotation for predicted genes using custom databases.

**1. Setup**

***1.1 Get raw sequence files***

*1.1a Test sample set [recommended option]:* We recommend an initial interactive test of the pipeline with two microbial samples. This will ensure that all necessary software is installed and that file paths are correct. A sample set can be downloaded using the command below:


mkdir raw_sequences # create directory for raw sequencescd raw_sequences # enter directorywget https://neon-microbial-raw-seq-files.s3.data.neonscience.org/2017/WOOD_002-M-20140925-comp_R1.fastq.gzwget https://neon-microbial-raw-seq-files.s3.data.neonscience.org/2017/WOOD_002-M-20140925-comp_R2.fastq.gzwget https://neon-microbial-raw-seq-files.s3.data.neonscience.org/2017/SCBI_012-M-20140915-comp_R1.fastq.gzwget https://neon-microbial-raw-seq-files.s3.data.neonscience.org/2017/SCBI_012-M-20140915-comp_R2.fastq.gzcd ..# return to enclosing directory


*1.1b Download custom dataset:* Use NEON’s interactive Data Portal, or to download a specific set of samples that meets your interests. Download links are included in NEON's “Expanded” data packages. For example, you could compare samples from Alaska with those from Puerto Rico, or you could download sites that have accompanying multi-decadal data from the Long-Term Ecological Research (LTER) program. Samples must have forward and reverse reads and they must be compressed (in.fastq.gz format). Even when compressed, each file may still require multiple GB of storage.

***1.2 Install Sunbeam***

Full details on Sunbeam installation can be found in the Sunbeam user guide. In short, run the following commands to create a new “analysis” directory and download Sunbeam into that directory:


mkdir metagenome_analysis # create directory for analysiscd metagenome_analysis # enter directorygit clone -b dev https://github.com/sunbeam-labs/sunbeam sunbeam # download development branchcd sunbeam # enter directorybash install.sh # run installation script


Confirm success of installation (may take 10-15 minutes):


bash tests/run_tests.bash


If all went well, your screen will say “TESTS SUCCEEDED.” A new conda environment should now exist. You can check available environments using:


conda env list


Activate the Sunbeam environment. This must be run for any Sunbeam commands to work.


conda activate sunbeam


Next, we tell Sunbeam where the raw sequences are downloaded, by creating a “samples.csv” file that links the forward read files and the reverse read files. If you have not downloaded files to a “raw_sequences” folder (Step 1.1A), change the file path to point to the sequence folder on your own system:


cd .. # go to enclosing (analysis) directorysunbeam list_samples ../raw_sequences >> samples.csv # change this path if your own raw files are not in “raw sequences”


The last part of setup requires creating a configuration file called “sunbeam_config.yml.” To use the custom configuration that accompanies this workflow run the following command from your analysis directory:


wget https://raw.githubusercontent.com/zoey-rw/metagenomes_NEON/main/sunbeam_config.yml # download configuration file


**Figure 1.**
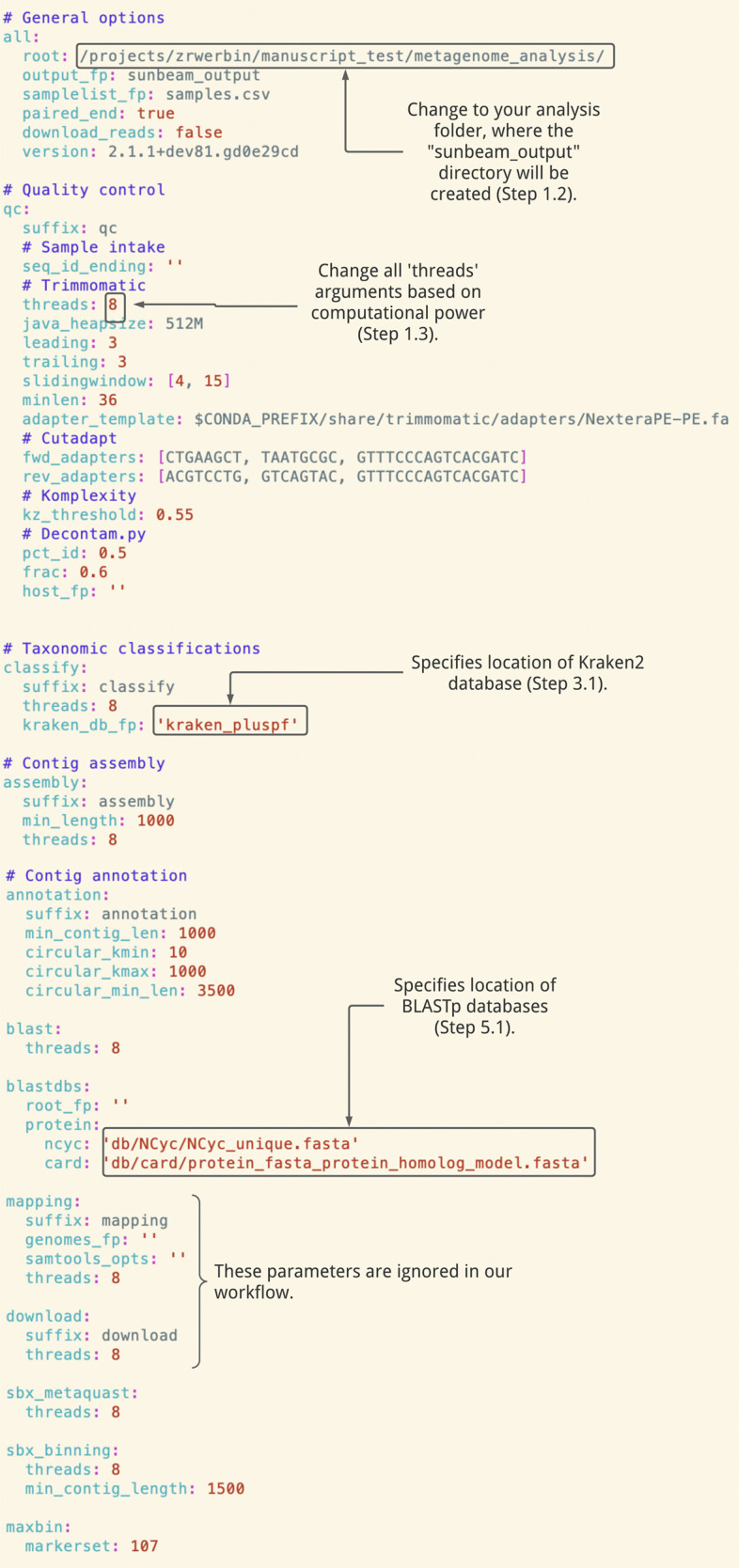
Sunbeam configuration file provided for NEON shotgun metagenomics bioinformatic pipeline. Many parameters remain the default values provided in Sunbeam’s basic configuration file, while others have been customized for this dataset (e.g. file paths, as well as fwd_adapter, rev_adapter, min_length).

This configuration file is used to set parameters for every part of the analysis ( Figure 1).

***1.3 Setup troubleshooting and tips***

On shared computing clusters, some softwares must be loaded as “modules” before they are used. For instance, to use Miniconda (necessary for every step of this pipeline), this command may work:


module load miniconda # may need to specify version


Most analyses will run quicker if there are multiple threads available. The custom configuration file, sunbeam_config.yml, assumes you have 8 threads available. This command can check your available threads, though you may not want to use all of them if you share computing resources:


echo "CPU threads: $(grep -c processor/proc/cpuinfo)"


**2. Quality control**

In this step, raw sequences are cleaned using the default tools in the Sunbeam pipeline. To remove poor-quality data, or components that are leftover from the sequencing, we use Cutadapt (Martin 2015) and Trimmomatic ( Bolger *et al.* 2014). Problematic low-complexity samples are removed using the program Komplexity ( Clarke *et al.* 2019). Overall quality of reads is then reported by FastQC (BabrahamBioinformatics, 2018).

**Figure 2.**
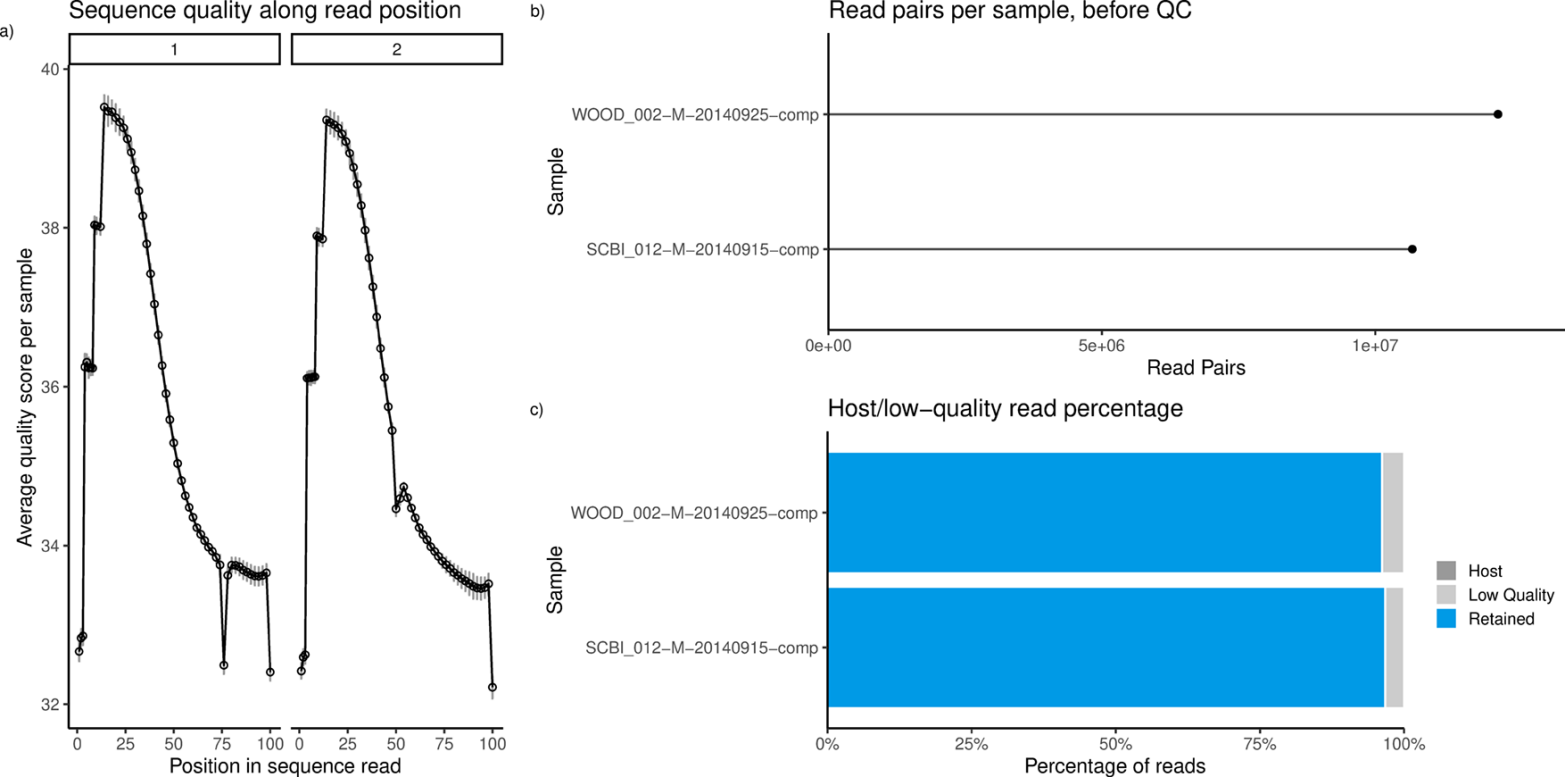
Quality-control reports produced by the *sbx_report* extension, described in Step 3.2A. a) Average quality scores along read positions. b) Counts of read pairs for a subset of samples. c) Proportion of reads retained (blue), discarded as low-quality (light grey), or discarded as PhiX (“Host”) contamination (dark grey). No PhiX contamination was observed in the metagenomes from these 2 NEON soil samples.

Optionally, users may wish to search for and remove sequences that match the PhiX genome (Step 2.1b), which is a common contaminant of Illumina metagenomic data due to its use as a control during sequencing ( Mukherjee *et al.* 2015). This contamination was not found in our test samples ( Figure 2c), so we proceed without this in Step 2.1a.

*2.1a Run quality control without PhiX decontamination [recommended]:* To run the quality control step without decontaminating the files, use the following command:


sunbeam run -- --configfile./sunbeam_config.yml clean_qc


*Note*: the below command does the same as the above, but produces intermediate outputs for each software (Cutadapt, Trimmomatic, and fastQC). This takes up additional file storage space, but allows you to inspect each output. This is useful for debugging, such as if you suspect that one of these steps is removing more reads than it should.


sunbeam run -- --configfile sunbeam_config.yml all_qc


*2.1b Run quality control with PhiX decontamination:* To download the PhiX genome, run the following command, which will retrieve the genome from the Illumina iGenomes website, decompress the file, and rename it as a FASTA file within your current directory:


wget http://igenomes.illumina.com.s3-website-us-east-1.amazonaws.com/PhiX/Illumina/RTA/PhiX_Illumina_RTA.tar.gz -O -|tar -xz PhiX/Illumina/RTA/Sequence/WholeGenomeFasta/genome.famv PhiX/Illumina/RTA/Sequence/WholeGenomeFasta/genome.fa PhiX/PhiX.fasta


In your configuration file, the “host_fp” parameter must point to the folder enclosing the downloaded PhiX genome. The command below will make this change:


sed -i "s/host_fp: “/host_fp: 'PhiX'/" sunbeam_config.yml


Next, run the Sunbeam decontamination step, which automatically includes quality control:


sunbeam run -- --configfile sunbeam_config.yml all_decontam


***2.2 Evaluate quality control***

Output folders contain log files for each software run within the quality control step. Each sample also has an HTML file produced by FastQC (BabrahamBioinformatics, 2018), which includes visualizations of base quality, sequence lengths, and other checks. More information on interpreting these reports is available on the FastQC website. By default, the samples with reads that pass quality control will be located in the following directory: sunbeam_output/qc/decontam/.

Within our example dataset, average quality scores were high (above 30) throughout sequence reads ( Figure 2a). Quality scores represent error rates of base calls ( Illumina, 2014). On average, the first few reads tended to be of lowest quality, but otherwise, quality decreases along read length. Quantity of sequences can vary dramatically between samples, with read pair counts ranging from 2 million to 15 million ( Figure 2b). This does not necessarily reflect variation in the amount of microbes in the soil - rather, variation can be the result of biases in DNA extraction or sequencing (Pereira *et al.* 2018; Jonsson *et al.* 2016).

**3. Taxonomic classification**

The taxonomic identity of reads in a metagenome sample can be assigned by comparing predicted proteins or nucleotides to reference databases. This can be performed with short reads (pre-assembly) or with assembled contigs. Both avenues produce similar results for fungal and bacterial sequences ( Pearman *et al.* 2020), so we use short reads for compatibility with Sunbeam’s default classifier, Kraken2 ( Wood *et al.* 2019). Compared to other classification tools, Kraken2 has been shown to perform favorably on soil datasets ( Kalantar *et al.* 2020). However, Sunbeam extensions have also been developed for other classifiers, such as Kaiju or MetaPhlAn.

***3.1 Classify reads using Kraken2***

First, we must download a Kraken2 reference database. You could build your own with specific combinations of organisms, but pre-built databases are updated regularly and shared by the Kraken2 developers. Databases range in size from 100 MB to 90 GB, depending on the genomes included. Most databases are constructed via RefSeq ( O’Leary *et al.* 2016), but marker gene databases such as Silva (Quast *et al.* 2012) and RDP ( Cole *et al.* 2014) may also be used with Kraken2.

Below, we use the “PlusPF” database, which includes sequences from archaea, bacteria, viral, plasmid, human, UniVec_Core, protozoa & fungi. The full database is 48 GB, but the version capped at 8 GB can be downloaded using this command:


wget -c https://genome-idx.s3.amazonaws.com/kraken/k2_pluspf_8gb_20210127.tar.gz -P kraken_pluspf/ # download databasetar -zxvf kraken_pluspf/k2_pluspf_8gb_20210127.tar.gz -C kraken_pluspf/ # decompress databaserm kraken_pluspf/k2_pluspf_8gb_20210127.tar.gz # remove compressed file


In your configuration file, the “kraken_db_fp” parameter should point to the folder enclosing the database ( Figure 1).

To run the taxonomic classification step:


sunbeam run -- --configfile sunbeam_config.yml all_classify


*3.2a Evaluate taxonomic classification using Sunbeam extension:* We can use a Sunbeam extension, *sbx_report* , to inspect results from the classification step. This will provide visual summaries of sequence quality along read position, read decontamination, and relative abundances of taxa from the phylum to the genus level. To download this extension, run:


sunbeam extend https://github.com/sunbeam-labs/sbx_report


**Figure 3.**
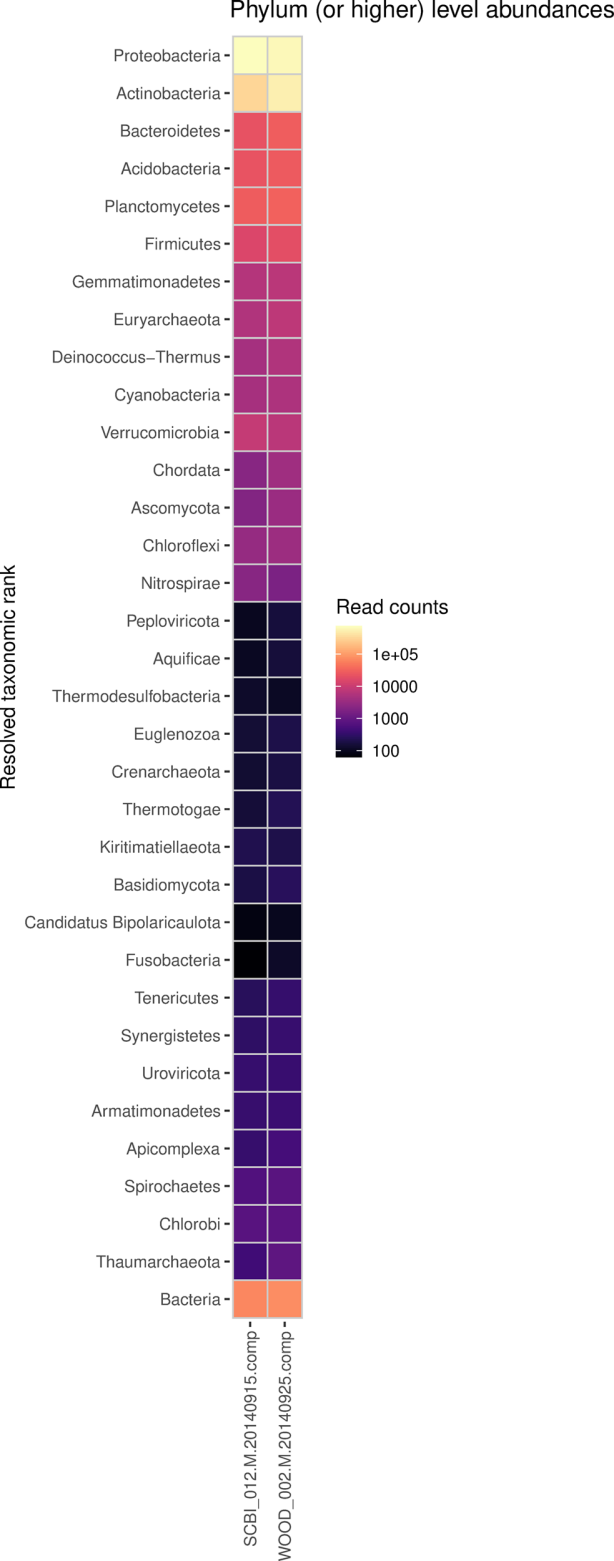
Taxonomic abundance reports produced by the *sbx_report* extension, described in Step 3.2A. Heatmap shows phylum-level read abundances for 2 NEON shotgun metagenomics samples.

Then run the following to generate HTML reports of read quality and taxonomic classification ( Figures 2 and 3):


sunbeam run -- --configfile sunbeam_config.yml --use-conda final_report

**4. Contig assembly**

This step takes the cleaned reads and assembles them into longer genome regions called contigs. We assemble reads into contigs to increase sensitivity and accuracy when predicting and annotating genes. Contig assembly has been shown to provide substantial improvements in conjunction with NCycDB in particular ( Anwar *et al.* 2019), which we use in Step 5. Contig assembly generally requires more computational power and time than any other step within metagenomic analysis ( Quince *et al.* 2017). Using multiple threads (i.e. 16) is recommended, and this may require adding the “--cores 16” argument to the Sunbeam command.

Below, we use the software Megahit ( Li *et al*. 2016), which is one of the fastest tools for metagenome assembly. For some samples, this speed may come at the expense of sensitivity, so users are welcome to substitute other software here. One option for this step is *co-assembly* of reads, in which information is shared between reads, which increases sensitivity to low-abundance reads ( Sczyrba *et al.* 2017). However, this causes an exponential increase in assembly time and memory usage, possibly taking days or weeks to complete.

*4.1a Assemble contigs independently [recommended option]:* In our configuration file ( Figure 1), we have set the minimum length of contigs to 1000bp using the ‘min_len’ parameter. This value represents the average gene length for prokaryotes ( Xu *et al.* 2006).


sunbeam run -- --configfile sunbeam_config.yml all_assembly


*4.1b Co-assemble contigs independently:* To take this route, you can use the extension shared by Sunbeam Labs, which carries out co-assembly using Megahit ( Li *et al*. 2016).

***4.2 Evaluate assembly output***

For each sample, basic summaries of the contig assembly are stored in the following directory by default: sunbeam_output/assembly/megahit/. Longer contigs generally represent higher confidence in longer regions of the genome, although misassemblies can occur and lead to long contigs ( Sczyrba *et al.* 2017). In the log files, you will find the minimum, maximum, and average contig length, as well as the number of contigs of at least 50bp.

**Figure 4.**
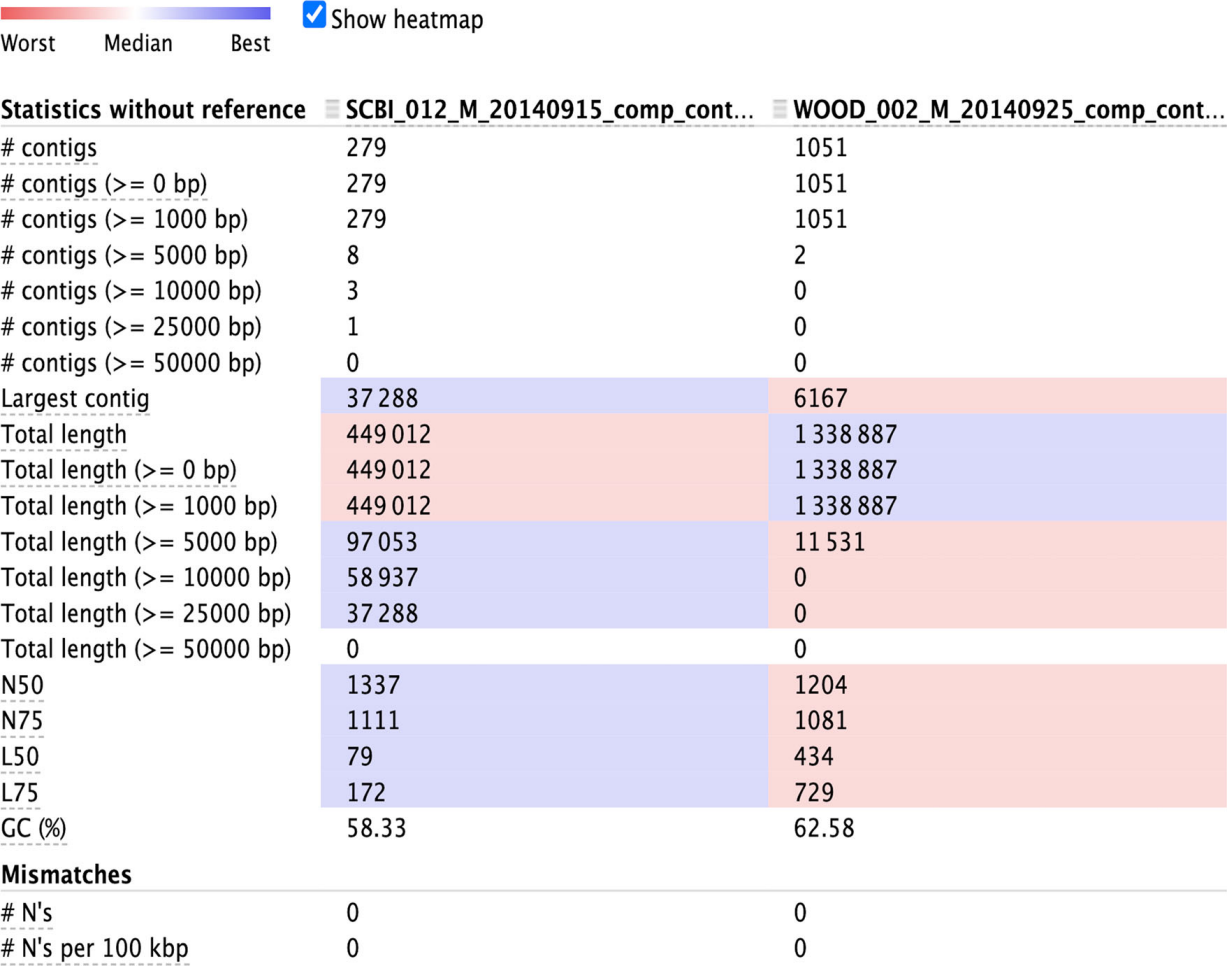
Output statistics from metaQUAST, summarizing contig lengths per sample. To produce similar statistics without downloading reference genomes, run metaQUAST with the “--max-ref-num” parameter set to 0.


*4.2a Optional: evaluate assembly output using metaQUAST:* We recommend the tool metaQUAST to perform a more in-depth evaluation assembly, such as summaries of contig length distributions ( Figure 4), detection of misassemblies and errors, or comparison with reference databases to estimate the abundance of unknown species ( Mikheenko *el al.* 2016). To download the metaQUAST program (as part of QUAST), run the following lines:

wget https://sourceforge.net/projects/quast/files/latest/download # download newest versiontar -xzf download # decompress file


To run the metaQUAST program on a sample or set of samples, specify the directory of input samples and output location like this (note: version number of QUAST may differ):


python./quast-5.0.2/metaquast.py -o metaquast_output/sunbeam_output/assembly/contigs/*.fa --max-ref-num 0


Section 2.4 of the metaQUAST manual discusses which reference genomes or databases are downloaded by default.

**5. Annotation**

The annotation step of the pipeline carries out BLAST searches on assembled contigs. Sunbeam will automatically use BLASTn for nucleotide databases, while BLASTx and BLASTp will be used for protein databases. Before protein databases are searched, the location of Open Reading Frames (ORFs) are predicted using the software Prodigal ( Hyatt *et al.*, 2010).

Gene presence does not necessarily mean that the genes are transcribed or active; however, due to the metabolically expensive nature of maintaining genomic pathways ( Lynch, 2006), there is potentially meaningful correspondence between gene presence and functional potential ( Pérez-Cobas *et al.* 2020). Below, we demonstrate preparation of two BLAST protein databases that may be scientifically relevant for soil metagenomics.

*Downloading the Comprehensive Antibiotic Resistance Database (CARD):* CARD ( Alcock *et al.* 2020) is a curated reference database of DNA sequences and proteins, designed to identify mutations and mechanisms of resistance to antibiotics, which can develop as a result of poor human stewardship ( Brown & Wright 2016). However, antibiotic resistance can also be an ecological signifier of fungal-bacterial competition for nutrients ( Bahram *et al.* 2018). We use the homolog protein genes to construct our reference database.


wget https://card.mcmaster.ca/download/0/broadstreet-v3.1.0.tar.bz2 -P db/card/ # download into new directorycd db/card/ # enter download directorytar -xf broadstreet-v3.1.0.tar.bz2./protein_fasta_protein_homolog_model.fasta # extract filecd ../../ # return to analysis directory


Next, we convert to BLASTp database for use within our pipeline:


makeblastdb -in db/card/protein_fasta_protein_homolog_model.fasta -title card_protein -dbtype 'prot' -hash_index # convert to BLASTp database


*Downloading NCycDB:* NCycDB categorizes genes into pathways that represent transformations such as nitrification, denitrification, and anammox. NCycDB was compiled from other sources, including COG, eggNOG, KEGG and the SEED ( Tu *et al.* 2019). The NCycDB must be downloaded from Github and converted into a BLAST protein database. From the analysis directory, run the following commands to download the database, decompress the file, and change the file suffix:


svn export https://github.com/qichao1984/NCyc/trunk/data/db/NCyc && gunzip db/NCyc/NCyc_100.faa.gz


This database has duplicate sequences that can introduce problems later on. We can remove duplicates using the following commands, which utilize the programs BLAST and cd-hit:


mv db/NCyc/NCyc_100.faa db/NCyc/NCyc_100.fasta # change file extensioncd-hit -i db/NCyc/NCyc_100.fasta -o db/NCyc/NCyc_unique.fasta -c 1 -t 1 # remove duplicate sequences


Next, we convert to BLASTp database for use within our pipeline:


makeblastdb -in db/NCyc/NCyc_unique.fasta -parse_seqids -title NCyc_unique -dbtype prot -hash_index # convert to BLASTp database


In your configuration file, the “root_fp” and “protein” parameters should point to the BLAST database directory and file names ( Figure 1). See the Sunbeam documentation for examples of configuration files that include nucleotide databases.

***5.1 Run annotation***

To run the annotation step:


sunbeam run -- --configfile sunbeam_config.yml all_annotate


**6. Annotation post-processing**

A suite of tools have been published for working with the BLASTxml outputs from Step 5. Python scripts can be used to convert BLASTxml to a CSV format; for examples, see the Github repository associated with this manuscript.

Once we have the read counts of genes associated with specific functions, we can compare results across samples. Gene counts should first be normalized to account for variation in sequencing depths (Pereira *et al.* 2018). One widely-used method is relative-log expression (RLE), which calculates scaling factors based on the geometric mean of gene abundances across samples. RLE can be implemented using the DESeq R package ( Love *et al.* 2014), and can be used to identify genes that are differentially abundant between groups (such as sites, or soil horizons).

**Figure 5.**
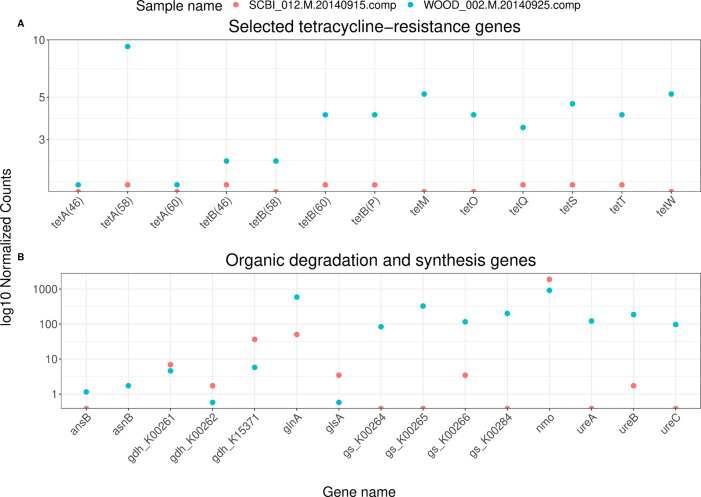
Log _10_ normalized counts from a BLASTp search of Open Reading Frames (ORFs) within contigs from two shotgun metagenomic samples. Contigs were assembled using Megahit ( Li *et al*. 2016), and ORFs were predicted using Prodigal ( Hyatt *et al.* 2010). These samples are a subset of the full NEON shotgun metagenomics dataset (NEON DP1.10107.001). A) BLASTp hits for a search against the Comprehensive Antibiotic Resistance Database (CARD) ( Alcock *et al.* 2020). Tetracycline resistance genes are defined as CARD entries with the word “tetracycline” in their description and “tet” in their name. B) BLASTp hits for a search against NCycDB ( Tu *et al.* 2019). Genes are subset to those belonging to “Organic degradation and synthesis” pathways.

For our two test samples, we can plot the outputs from each BLASTp search ( Figure 5). Among antibiotic resistance genes, we can look at trends for specific types of antibiotics. Tetracycline resistance, for example, has become widespread in soil bacteria, possibly linked to intensive farming ( Schmitt *et al.* 2006). For a subset of tetracycline-resistance genes, normalized abundances appear higher in the sample from the NEON’s WOOD site ( Figure 5A). For our nitrogen-cycling genes, we can subset to those associated with organic synthesis and degradation. For these genes, we see a similar pattern, with higher normalized abundances in the sample from the WOOD sites ( Figure 5B). However, the SCBI sample had a lower sequencing depth overall ( Figure 2B), which can prevent the detection of low-abundance genes.

**7. Exporting to KBase for binning**

The outputs from this pipeline can be further analyzed using the KBase platform, developed by the U.S. Department of Energy for microbiome analysis ( Arkin *et al.* 2018). KBase links hundreds of different software tools using an online interface, which allows users to create “Narratives” for specific data analysis projects. Individual files can be uploaded to KBase directly, or they can be transferred in batches using Globus Online ( Foster 2011).

**Figure 6.**
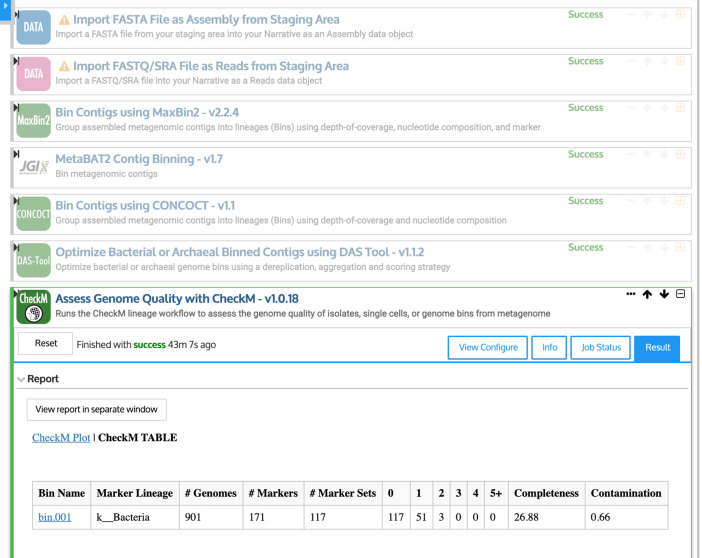
Creating and evaluating Metagenome-Assembled Genomes (MAGs) using the KBase Narrative interface ( Arkin *et al.* 2018). First, quality-controlled sequencing reads and assembled contigs are imported using upload modules. Then, contigs are binned into putative genomes (or “bins”) using MaxBin2 ( Wu *et al.* 2016), MetaBAT2 ( Kang *et al.* 2019), and CONCOCT ( Alneberg *et al.* 2014). Finally, DAS Tool ( Sieber *et al.* 2018) is used to choose the highest-quality bins.

For example, a KBase Narrative ( Figure 6) could be used to create Metagenome-Assembled Genomes (MAGs). Because MAGs are created directly from contigs, rather than from microbes grown in an experimental setting, they often have no cultured relatives, representing a hidden source of genetic diversity in the microbiome ( Nayfach *et al.* 2020). KBase includes a variety of tools for creating MAGs, each using different algorithms, and outputs from multiple tools can be synthesized using a program called DAS Tool ( Sieber *et al.* 2018). For each putative genome, or “bin,” summary statistics are produced that estimate the completeness and possible contamination of the genome, using a set of genes that are expected to be “single-copy” within a genome ( Sieber *et al.* 2018).

In our example Narrative, we combine the output from three tools, MaxBin2 ( Wu *et al.* 2016), MetaBAT2 ( Kang *et al.* 2019), and CONCOCT ( Alneberg *et al.* 2014). As inputs, we use the contigs assembled in Step 4 of this pipeline, as well as the quality-controlled sequencing reads from Step 2, for the sample WOOD_002-M-20140925-COMP. For this sample, DAS Tool produces one genome, *bin.001,* which is less than 27% complete. Bins can be further refined manually, and genomes that are more than 90% complete with less than 5% contamination may be good candidates for submission to public databases (Bowers *et al.* 2017). High-quality MAGs can uncover entirely new lineages in the microbial tree of life ( Nayfach *et al.* 2020).

**Troubleshooting, tips and tricks**

For any rule, if not all files are processed, the step can be repeated using the --unlock and --rerun-incomplete parameters, i.e.:


sunbeam run -- --configfile sunbeam_config.yml clean_qc --rerun-incomplete --unlocksunbeam run -- --configfile sunbeam_config.yml clean_qc --rerun-incomplete


To customize or expand on the workflow above, it is helpful to know the basic logic of Snakemake, which is the underlying framework for the Sunbeam pipeline. Snakemake relies on a series of rules, which specify input files, output files, and any necessary commands. When a rule is called, Snakemake works backwards from the output files to decide if any input files are missing or outdated, and tries to re-run rules as needed. If you want to add an extension to Sunbeam, a full guide is available in the Sunbeam documentation.

To scale up to a larger dataset, a significant amount of computational power will be necessary, ideally with 8 or more cores for parallel computation. For those without access to institutional high-performance clusters, the scientific computing platform CyVerse (Merchant *et al.* 2016) offers free computational and storage resources. Note that intermediate files are generated for multiple steps, which can multiply the amount of storage needed for each metagenomic sample. Deleting these intermediate files when a step has completed will reduce the storage requirements.

## Data availability

Raw metagenomics sequencing data is published as DP1.10107.001 from the National Ecological Observatory Network ( https://data.neonscience.org/data-products/explore). All other data is previously published and cited throughout the paper.

## Software availability

Bioconductor packages available at https://www.bioconductor.org/. CRAN packages available at https://cran.r-project.org/. Sunbeam software available at https://sunbeam.readthedocs.io.

Scripts to download NEON raw data, as well as process final BLASTxml files, are hosted at https://github.com/zoey-rw/metagenomes_NEON.

Archived scripts as at time of publication: http://doi.org/10.5281/zenodo.4589528 ( Werbin 2021).

License: Creative Commons Zero v1.0 Universal.
