# The National Ecological Observatory Network’s soil metagenomes: assembly and basic analysis

**DOI:** 10.12688/f1000research.51494.2

**Published:** 2022-03-23

**Authors:** Zoey R. Werbin, Briana Hackos, Jorge Lopez-Nava, Michael C. Dietze, Jennifer M. Bhatnagar

**Affiliations:** 1 Department of Biology, Boston University, Boston, MA, 02215, USA; 2 Department of Mathematics, University of Colorado, Boulder, Boulder, CO, 80309, USA; 3 Department of Mathematics, Swarthmore College, Swarthmore, PA 19081, USA; 4 Department of Earth & Environment, Boston University, Boston, MA, 02215, USA

**Keywords:** metagenomics, microbial ecology, soil microbiome, tutorial, workflow

## Abstract

The largest dataset of soil metagenomes has recently been released by the National Ecological Observatory Network (NEON), which performs annual shotgun sequencing of soils at 47 sites across the United States. NEON serves as a valuable educational resource, thanks to its open data and programming tutorials, but there is currently no introductory tutorial for accessing and analyzing the soil shotgun metagenomic dataset. Here, we describe methods for processing raw soil metagenome sequencing reads using a bioinformatics pipeline tailored to the high complexity and diversity of the soil microbiome. We describe the rationale, necessary resources, and implementation of steps such as cleaning raw reads, taxonomic classification, assembly into contigs or genomes, annotation of predicted genes using custom protein databases, and exporting data for downstream analysis. The workflow presented here aims to increase the accessibility of NEON’s shotgun metagenome data, which can provide important clues about soil microbial communities and their ecological roles.

## Introduction

The soil microbiome is responsible for key ecological processes, such as decomposition and nitrogen cycling ( Allison *et al.*, 2013). One powerful tool for studying the soil microbiome is shotgun metagenomic sequencing, in which all of the genetic material within the DNA extract of a soil sample is sequenced at once, without targeting specific organisms ( Quince *et al.*, 2017; Pérez-Cobas *et al.*, 2020). The largest publicly available sequencing dataset of this type is updated annually by the National Ecological Observatory Network (NEON), which monitors ecological conditions at 47 terrestrial sites spanning 20 ecoclimatic domains across the US and its territories ( Keller *et al.*, 2008). NEON is funded by the National Science Foundation (NSF), and collects soil samples and releases shotgun metagenomics data annually.

To date, the NEON soil metagenomics data can only be accessed in two formats: as completely raw reads released by NEON, or as processed files through the default protocols of the MG-RAST storage server. Neither format is suitable for most metagenomic analyses, which generally answer scientific questions using custom data processing pipelines that use specific algorithms and targeted reference databases ( Ladoukakis *et al.*, 2014; Quince *et al.*, 2017). However, the hyperdiversity of soil ecosystems can pose a challenge for even the most cutting-edge genomic software: retrieving complete bacterial genomes is especially difficult from soil samples ( Sieber *et al.*, 2018), and up to 95% of soil DNA reads cannot be identified to the genus level ( Méric *et al.*, 2019). To facilitate future scientific analysis, we present a workflow for taking raw soil sequences and generating a processed dataset that can be linked to other NEON data products, which include soil biogeochemistry, root measurements, or aboveground plant communities.

NEON data is a valuable resource for ecology and bioinformatics, thanks to its open access software, robust documentation, and educational resources ( Jones, 2020). The pipeline that we present here is designed to complement existing NEON educational resources, such that students and researchers with basic bioinformatics experience may use this dataset to learn about microbial communities within the soil. We present code and explanations for common analysis steps, including basic quality control (QC), assembling reads into larger genome fragments (“contig” assembly), predicting genes, quantifying gene counts for specific ecological or biogeochemical functions, genome assembly, and exporting to the KBase platform ( Arkin *et al.*, 2018). We recommend the review by Pérez-Cobas *et al.*, (2020) for software alternatives for each step of this shotgun metagenomics analysis.

## Methods

### Dataset description

Soil samples are collected annually from 47 NEON sites during peak greenness. Soil samples are collected up to 30cm below the soil surface, the organic (O) and the mineral (M) horizons (when present) are separated, and subsamples from each horizon are homogenized into one composite sample per horizon, and frozen on dry ice until DNA extraction. Sample file names include the 4-letter site identifiers, soil horizons (O or M), sampling date, and replicate number. Three samples are collected within a NEON plot at a sampling time point. As of 2021, DNA extractions are performed using KAPA Hyper Plus kit (Kapa Biosystems). Samples from multiple sites are pooled into sets of 40 or 60 for 150 bp paired-end sequencing, which is conducted on an Illumina NextSeq at the Battelle Memorial Institute (NEON Metagenomics Standard Operating Procedure, v.3). While there is currently no versioned release of NEON’s metagenomic data, the pipeline described here is designed to be robust to processing new short-read sequence data as they are released from NEON, approximately annually, though protocols may shift over NEON’s 30-year time span ( Stanish & Parnell, 2018).

### Operation

We assume a Linux operating system and command-line interface. Storage and RAM requirements will depend on the specific analyses performed and the number of samples analyzed. To work with a large dataset (10+ samples), a significant amount of computational power will be necessary, ideally with 8 or more cores for parallel computation. For those without access to institutional high-performance clusters, the scientific computing platform CyVerse ( Merchant *et al.*, 2016) offers free computational and storage resources.

The computing requirements for metagenomic analysis can sometimes overwhelm personal computers, or login nodes on shared computing clusters. Therefore, users may wish to test the pipeline in a local environment, then shift to a high-performance cluster for large numbers of samples. Due to the long duration of certain steps, users may benefit from Linux commands that prevent sessions from timing-out or dropping the connection, such as *tmux* or *screen* . Either method requires modifying the configuration file called “config.yaml.” **Bolded text will be used to emphasize parameters that should be modified within the configuration file.**

*Local analysis:* Each metaGEM command can be run with a “--local” flag to run within your current environment. If you have access to multiple cores, then you will need to add the “--cores” flag to each metaGEM commands below, to take advantage of parallel computing. This command can check your available threads, though you may not want to use all of them if you share computing resources:

echo "CPU threads: $(grep -c processor/proc/cpuinfo)"


*Cluster analysis:* To run on a cluster, the pipeline will assume that jobs are submitted via a SLURM-based scheduling system, controlled using the file called “cluster_config.json.” Clusters with SGE/OGE-based scheduling may require workarounds. **The “cores” section of the configuration file should be modified to reflect the number of computing cores for each step.** Contact your system administrator for information on appropriate scratch directories, or for guidance on scheduling and configuration files.

On shared computing clusters, some softwares must be loaded as “modules” before they are used. For instance, to use Miniconda (necessary for every step of this pipeline), this command will work if there is a shared installation:

module load miniconda # may need to specify version


If there is no existing Miniconda installation, follow the instructions from Conda for a new installation. Subsequent code will assume that analysis is running locally within a Miniconda environment.

### Implementation

Once sequences are downloaded, we use the pipeline metaGEM ( Zorrilla *et al.*, 2021), which links a variety of bioinformatics tools and users can develop customized extensions for specific purposes. metaGEM, and its underlying Snakemake framework ( Köster & Rahmann, 2012), are designed to address common problems with software versioning and updating, as well as efficient data re-analysis (i.e. running the minimal tasks necessary to generate updated output files). We describe installation and use instructions for metaGEM below. In addition to metaGEM default steps for cleaning and assembling the raw reads, we describe taxonomic classification or protein annotation for predicted genes using custom databases.

To customize or expand on the workflow below, it is helpful to know the basic logic of Snakemake, which is the underlying framework for the metaGEM pipeline. Snakemake relies on a series of rules, which specify input files, output files, and any necessary commands. When a rule is called, Snakemake works backwards from the output files to decide if any input files are missing or outdated, and tries to re-run rules as needed ( Köster & Rahmann, 2012).

### Setup: installing metaGEM pipeline

Full details on installation can be found in the metaGEM wiki. In short, run the following commands to create and setup a new analysis directory called metaGEM:

git clone https://github.com/franciscozorrilla/metaGEM.git # Download metaGEM repo
cd metaGEM # enter directory
bash env_setup.sh # Run automated setup script


Confirm success of installation and environment setup:

bash metaGEM.sh -t check


If all went well, your screen will report messages about the installation. Otherwise, it will report any problems in specific package installations or environments. You can inspect at the new environments using:

conda env list


Activate the metaGEM conda environment. This will be used for most parts of the pipeline.

conda activate metaGEM


Open the configuration file called “config.yaml” and modify paths as needed. **Users must specify the location for the analysis environment, as well as a “scratch” directory for temporary files.**

## 1. Accessing raw sequence files

### 1.1 Download test dataset

We recommend an initial interactive test of the pipeline with two microbial samples. This will ensure that all necessary software is installed and that file paths are correct. From within the metaGEM directory, a sample set can be downloaded using the code block below:

cd dataset # enter data directory (within metaGEM directory)
wget https://neon-microbial-raw-seq-files.s3.data.neonscience.org/2017/WOOD_002-M-20140925-comp_R1.fastq.gz
wget https://neon-microbial-raw-seq-files.s3.data.neonscience.org/2017/WOOD_002-M-20140925-comp_R2.fastq.gz
wget https://neon-microbial-raw-seq-files.s3.data.neonscience.org/2017/SCBI_012-M-20140915-comp_R1.fastq.gz
wget https://neon-microbial-raw-seq-files.s3.data.neonscience.org/2017/SCBI_012-M-20140915-comp_R2.fastq.gz
cd ..# return to enclosing metaGEM directory


Next, we have metaGEM reorganize the raw sequence files into subfolders.

bash metaGEM.sh --task organizeData


### 1.2 Download custom dataset

Information about the metagenomic sequencing for each soil sample is contained in the NEON data product DP1.10107.001, which can be accessed using the interactive Data Portal.

Data from specific sites and dates can also be accessed via the neonUtilities R package ( Lunch *et al.*, 2021). The R commands below will download the DP1.10107.001 metadata for all samples collected from the Harvard Forest site in the year 2018. This metadata can then be used to download raw sequences.

# install neonUtilities - can skip if already installed
install.packages("neonUtilities")
# load neonUtilities
library (neonUtilities)
metadata <- loadByProduct (dpID = 'DP1.10107.001', site="HARV", startdate = "2018-01", enddate = "2018-12", package = 'expanded')


Downloads will come with three tables of interest:

• mms_metagenomeDnaExtraction: reports the quantity of DNA extracted from the soil sample.
• mms_metagenomeSequencing: lists sequencing protocol for each sample, as well as the read counts. These read counts can be used to filter out low-quality samples.
• mms_rawDataFiles: lists the download URL for each sample. This table is included only with the “expanded” package setting, not the “basic” setting.

The sites and dates of interest should be determined by the goals of your analysis: a comparative study might require samples from Alaska as well as from Puerto Rico, or samples could be retrieved from sites that have accompanying multi-decadal data from the Long-Term Ecological Research (LTER) program. If samples have the extension.tar.gz, then they are bundled into a compressed folder with multiple samples and will need to be unbundled (see tutorial here). Samples must have forward and reverse reads and they should be compressed in.fastq.gz format for most downstream software. Even when compressed, each file may still require multiple GB of storage.

## 2. Quality control

### 2.1 Background and rationale

Raw sequences are shared online in FASTQ format, with only minimal quality control from NEON’s sequencing facilities, since users may prefer to use specific protocols for quality control. Some aspects of quality control present a trade-off between data volume and data quality. Each base returned by a sequencing machine (e.g. “A”, “C”, “T”, or “G”) has an associated quality score, or *Q score* ( Cock *et al.*, 2009). Q scores can be used to filter low-quality reads, which generally improves the reliability of genomic analysis ( Illumina, 2014). Certain aspects of quality control are absolutely necessary for reliable analysis, such as removing adapter or primer sequences used in sequencing protocols. For these steps, Cutadapt ( Martin, 2010) and Trimmomatic ( Bolger *et al.*, 2014) are frequently-used tools and work well. Fastp ( Chen *et al.*, 2018) is an all-in-one QC tool included in the metaGEM pipeline (Section 2.3) ( Zorrilla *et al.*, 2021).

Optional steps of quality-control include removing low-complexity sequences and searching for contaminants. Low-complexity sequences are naturally occurring regions of DNA with highly biased distributions of bases, such as “AAAAAAAAAGCGCTTTTTTT.” These regions can make matching to gene databases more difficult by causing spurious results ( Clarke *et al.*, 2019). Users may wish to search for and remove contaminant sequences, such as those that match the PhiX genome, which is a common contaminant of Illumina metagenomic data due to its use as a control during sequencing ( Mukherjee *et al.*, 2015).

### 2.2 Considerations for NEON data

**Figure 1.**
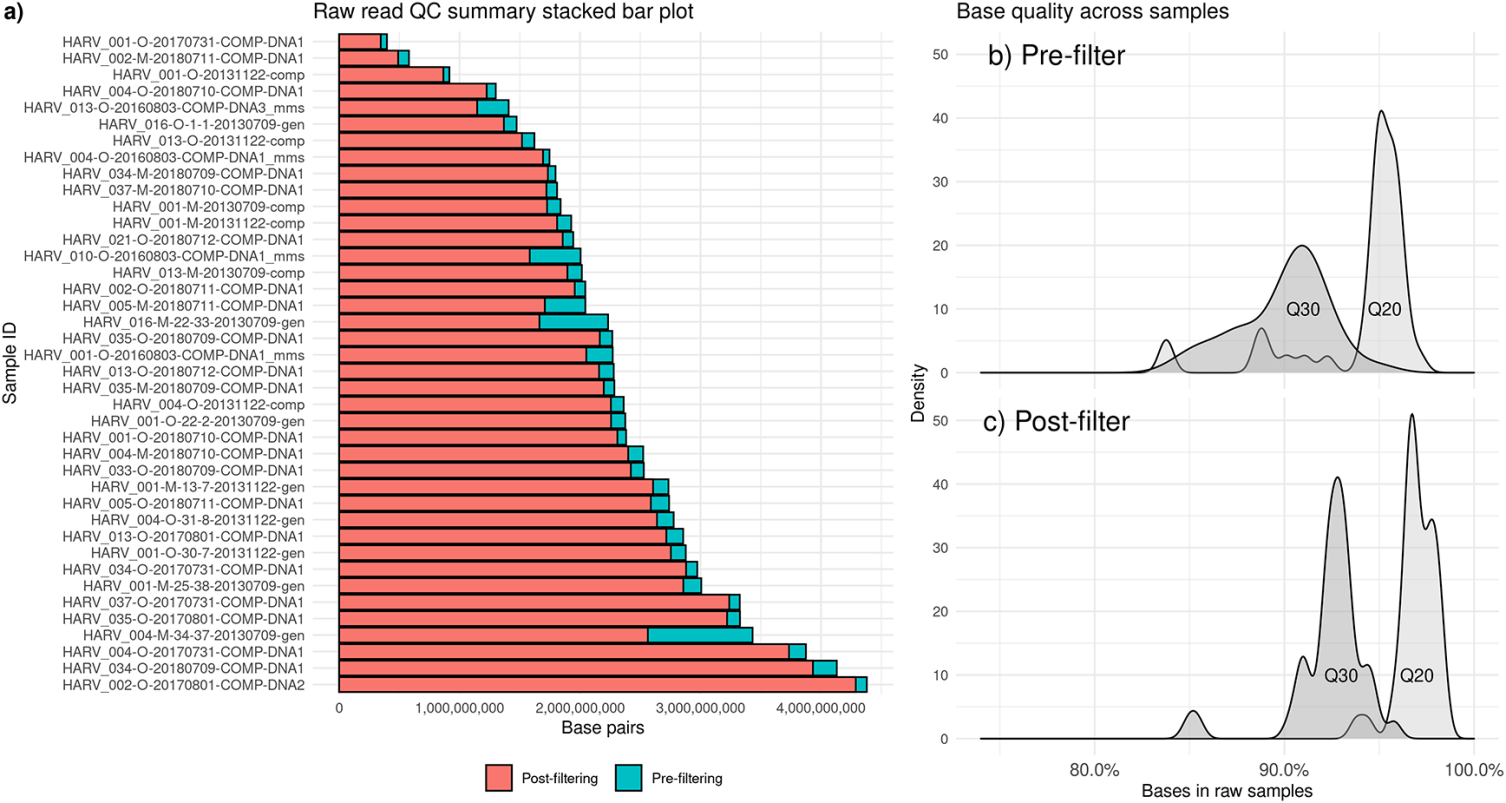
Quality control results for short reads using the Fastp software ( Chen *et al.*, 2018). Short-read metagenomic samples are from the Harvard Forest site of the National Ecological Observatory Network (NEON). a) Counts of read pairs before (blue) and after (red) quality control steps. b) Base quality at Q30 (dark gray) and Q20 (light gray) before filtering. c) Base quality at Q30 (dark gray) and Q20 (light gray) after filtering.

Soil samples from NEON have a wide range of average quality scores, as well as a range of sequencing depths, which are affected by DNA amounts in soil, lab DNA extraction efficiency, and sequencer error. We recommend removing samples with lower sequencing depths, but the specific depth cutoff will vary based on your analysis goals ( Brumfield *et al.*, 2020). Up to 100 Gbp may be required for characterizing full soil diversity ( van der Walt *et al.*, 2017). None of NEON’s metagenomes meet this ultra-high sequencing depth, but the majority are sequenced to at least 1.5 Gbp ( Figure 1a).

In a subset of NEON metagenomes, we did not find PhiX contamination, so this step is not implemented in Section 2.3. However, tools for removing low-complexity sequences (Komplexity) and removing contaminant DNA are included in the Sunbeam pipeline ( Clarke *et al.*, 2019), an alternative to the metaGEM pipeline used throughout.

### 2.3 Implementation via metaGEM pipeline

To run quality control on raw sample files (primer trimming, adapter trimming, read filtering, and base quality evaluation) run the following command:

bash metaGEM.sh --task fastp --local


Each sample will have detailed report files within the “qfiltered” directory. To summarize the results across all samples, run the following command:

bash metaGEM.sh --task qfilterVis --local


Simple visualization of QC outputs will then be generated within the “stats” directory.

## 3. Assembly-free analysis

### 3.1 Background and rationale

Metagenomic analysis often involves assembling short reads into longer fragments, called contigs, which can be searched for genes. However, the assembly step is computationally intensive, and may be avoidable if the only desired output is a taxonomic profile, which can be generated by tools designed to work with unassembled short reads ( Pearman *et al.*, 2020). These tools, such as Kraken2 ( Wood *et al.*, 2019) or Kaiju ( Menzel *et al.*, 2016), can assign taxonomic identities to reads by comparing sequences to reference databases. Compared to other classification tools, Kraken2 has been shown to perform favorably on soil datasets ( Kalantar *et al.*, 2020; Lu & Salzberg 2020). However, the vast majority of soil reads remain unclassified with short-read classifiers. This may be due to the lack of complete genomes from soil organisms within reference databases ( Quince *et al.*, 2017).

### 3.2 Considerations for NEON data

Taxonomic reference databases can include sequences from various biological domains, often using genomes from RefSeq ( O’Leary *et al.*, 2016) or marker gene databases such as Silva ( Quast *et al.*, 2013) and RDP ( Cole *et al.*, 2014). The “Standard” pre-built database, shared by the Kraken2 developers, contains sequences from archaea, bacteria, viral, plasmid, human, and UniVec_Core. Due to the importance of fungi within soil ecosystems, we tested a larger database (“PlusPF”) that also includes fungi and protozoa. Overall, approximately 17% of reads were identifiable to any kingdom, with fewer than 0.1% assigned to fungi. Given the increased memory costs of larger databases, and the low detection of fungi and protozoa, a smaller database (e.g. the Standard) is likely preferable for most microbial analyses. Other NEON microbial data products (such as amplicon sequences, qPCR, and PLFA) can provide domain-specific information on fungi, bacteria, and archaea.

### 3.3 Implementation

The Kraken2 reference databases that span multiple domains of life can reach 100 gigabytes, presenting a potential obstacle to running analyses on personal computers. The Toolchest R package ( Cai & Lebovic, 2021) allows for remote Kraken2 analysis of samples from within the R environment. The example code below uses the “PlusPF” Kraken2 database, which includes sequences from archaea, bacteria, viral, plasmid, human, protozoa, fungi, and vector contaminants. Results for each sample are summarized in a “report” file, which sums the number of reads assigned to each taxon.

install.packages("toolchest")
library("toolchest")
toolchest::set_key("share.NjYyZDE2ZTUtNTU0Ny00OWQzLTlkNTktYjRmMTAzYmM4NWFh") # example key with limited capacity - please download a new key from the Toolchest website
kraken2(read_one = "WOOD_002-M-20140925-comp_R1.fastq.gz",
     read_two = "WOOD_002-M-20140925-comp_R2.fastq.gz",
     output_path = "./kraken_output.txt")


Kraken2 report files can be visualized using the software Pavian ( Breitwieser & Salzberg, 2020). Pavian can be run locally via R, or samples can be uploaded for analysis using the online application. Alternatively, output from Kraken2 can be converted to the BIOM file format for in-depth visualization using the metagenomics exploration software Phinch ( Bik, 2014).

## 4. Contig assembly

### 4.1 Background and rationale

Assembling short reads into contigs can increase sensitivity and accuracy when predicting and annotating genes. Contig assembly generally requires more computational power and time than any other step within metagenomic analysis ( Quince *et al.*, 2017).

Assembly of soil metagenomes is particularly difficult due to high amounts of biodiversity per sample and the absence of organisms in reference databases. Currently, the only assembly software designed for soils is Megahit ( Li *et al.*, 2016), which is also one of the fastest tools for metagenome assembly. For some samples, this speed may come at the expense of sensitivity. metaSPAdes has been benchmarked with soil data and performs comparably, sometimes producing longer contigs, but requires additional memory and runtime ( van der Walt *et al.*, 2017).

*Co-assembly* of reads, in which information is shared between samples, increases sensitivity to low-abundance reads ( Sczyrba *et al.*, 2017), and can aid in recovering rare genomes ( Albertsen *et al.*, 2013). However, co-assembly causes an exponential increase in assembly time and memory usage, possibly taking days or weeks to complete. Co-assembly can also increase the number of chimeric contigs for samples with high strain diversity ( Ramos-Barbero *et al.*, 2019).

Other assembly decisions (such as minimum contig length) should depend on downstream analyses; for example, average prokaryotic genes are about 1000 bp ( Xu *et al.*, 2006), so shorter contigs may not contain useful information on gene presence or absence. Some genome binning tools, such as metaBAT, will discard any contigs lower than 1500 bp. Very low thresholds, such as 300 or 500 bp, will increase the percentage of raw reads that are represented in an assembly. Longer contigs generally represent higher confidence in longer regions of the genome, although misassemblies can occur and lead to long contigs ( Sczyrba *et al.*, 2017). We recommend the tool metaQUAST to perform in-depth evaluation assembly, such as summaries of contig length distributions, detection of misassemblies and errors, or comparison with reference databases to estimate the abundance of unknown species ( Mikheenko *et al.*, 2016). The review by Ayling *et al.* (2020) covers recent developments in short-read assembly approaches and reference-free assembly evaluation.

### 4.2 Considerations for NEON data

The variation in sequencing depth among NEON soil samples corresponds to high variation in assembly length ( Figure 3A). Samples with deeper sequencing depths had, on average, longer contig lengths ( Figure 3B). Most assemblies consisted of thousands of separate contigs ( Figure 3C). Due to the effort required for assembly, it may be preferable to select a subset of high-quality samples for downstream analysis, rather than assembling all samples.

Co-assembly of samples may improve assemblies, but it is currently unclear how samples should be grouped for optimal results, since co-assembly can improve some aspects of an assembly while also introducing errors ( Ramos-Barbero *et al.*, 2019). Some options include grouping samples by sampling plot, timepoint, soil horizons, or field site.

### 4.3 Implementation

For the contig assembly step, we recommend changing certain parameters in the configuration file. Under the “params” section, the assemblyPreset parameter is passed to the assembly software, Megahit. **The default value is “meta-sensitive”, but the “meta-large” setting is optimized for complex soil datasets.**

To assemble contigs, run the following command, specifying the number of available cores:

bash metaGEM.sh --task megahit --local --cores 28
bash metaGEM.sh --task assemblyVis


Visualization of assembly outputs are also located within the “stats” subfolder.

## 5. Functional gene annotation

### 5.1 Background and rationale

To estimate the functional capabilities of a soil microbial community, gene annotation can be carried out using various gene reference databases. This annotation step can be performed on short reads (i.e. the output from the quality filtering steps), but this can lead to false positives due to short reads matching multiple ambiguous regions of reference genes ( Quince *et al.*, 2017). More confident matches can often be obtained by searching for genes within assembled contigs. However, soils often have low assembly rates, in which only a small portion of reads end up as part of a contig ( Vollmers *et al.*, 2017), which can skew functional profiles. The benefit of assembling before annotation can be diminished if fewer than 85% of reads map to contigs ( Tamames *et al.*, 2019).

Functional gene annotation of unassembled reads is carried out for all NEON samples on MG-RAST at the time of their online publication, using a collection of functional gene databases such as eggNOG ( Huerta-Cepas *et al.*, 2019), KEGG ( Kanehisa *et al.*, 2017), and SwissProt ( Boutet *et al.*, 2007). Gene annotation from multiple databases can dramatically increase the number of annotated genes, a trend that is especially pronounced for microbes (such as soil organisms) that are only distantly related to model organisms like *E. coli* ( Griesemer *et al.*, 2018).

When annotating genes in assembled contigs, a preliminary step is to identify Open Reading Frames (ORFs) using software such as Prodigal ( Hyatt *et al.*, 2010). Then, BLASTp ( Altschul *et al.*, 1990) or DIAMOND2 ( Buchfink *et al.*, 2021) can be used to search against protein gene databases. Gene presence does not necessarily mean that the genes are transcribed or active; however, due to the metabolically expensive nature of maintaining genomic pathways ( Lynch, 2006), there is potentially meaningful correspondence between gene presence and functional potential ( Pérez-Cobas *et al.*, 2020).

### 5.2 Considerations for NEON data

Soil metagenomes can be used to explore functions of biogeochemical, medical, or ecological interest. For example, the Comprehensive Antibiotic Resistance Database (CARD) ( Alcock *et al.*, 2020) is a curated reference database of DNA sequences and proteins, designed to identify mutations and mechanisms of resistance to antibiotics, which can develop as a result of poor human stewardship ( Brown & Wright 2016). However, antibiotic resistance can also be an ecological signifier of fungal-bacterial competition for nutrients ( Bahram *et al.*, 2018). Another protein database with relevance to the soil microbiome is NCycDB, which categorizes genes into pathways that represent transformations such as nitrification, denitrification, and anammox. NCycDB was compiled from other sources, including COG, eggNOG, KEGG and the SEED ( Tu *et al.*, 2019).

While functional gene profiling is more reliable with contigs rather than short reads ( Anwar *et al.*, 2019), we note that only 5-10% of reads mapped to any contigs within select Harvard Forest samples (minimum contig length 1000, and pseudoalignment carried out using Kallisto with default settings ( Bray *et al.*, 2016)). These low mapping rates may suggest that our assembled contigs represent only a small portion of the soil metagenome.

### 5.3 Implementation

For this example, we will search samples for genes from NCycDB. NCycDB has been shown to return fewer false positives when used with assembled contigs rather than unassembled short reads ( Anwar *et al.*, 2019), so the following steps use the assembled contigs as input.

The NCycDB must be downloaded from Github and converted into a BLAST-compatible protein database. From the metaGEM directory, run the following commands to download the database:

svn export https://github.com/qichao1984/NCyc/trunk/data/NCyc_100_2019Jul.7z db/NCyc_100_2019Jul.7z


This file must be decompressed from “7z” format into “.faa” format. Commands for this will vary based on your operating system.

Next, we use the program Diamond ( Buchfink *et al.*, 2021) to convert to BLAST-compatible database for use within our pipeline:

diamond makedb --in db/NCyc_100_2019Jul.faa -d db/NCyc_DB


**In your configuration file, the “blast_db” parameter should be modified to point to the database file name.**

To predict the genes on the assembled contigs, run Prodigal via the following command:

bash metaGEM.sh --task run_prodigal


To compare the predicted genes with the NCycDB, run the following command:

bash metaGEM.sh --task run_blastp


To interpret the output files, each gene can be linked to its gene family using the “id2map” file associated with NCycDB:

svn export https://github.com/qichao1984/NCyc/trunk/data/id2gene.map.2019Jul db/id2gene.map.2019Jul


To compare results across samples, gene counts must be normalized to account for variation in sequencing depths ( Pereira *et al.*, 2018). One widely-used method is relative-log expression (RLE), which calculates scaling factors based on the geometric mean of gene abundances across all samples. RLE can be implemented using the DESeq R package ( Love *et al.*, 2014), and can be used to identify genes that are differentially abundant between groups (such as field sites, or soil horizons).

## 6. Binning

### 6.1 Background and rationale

**Figure 2.**
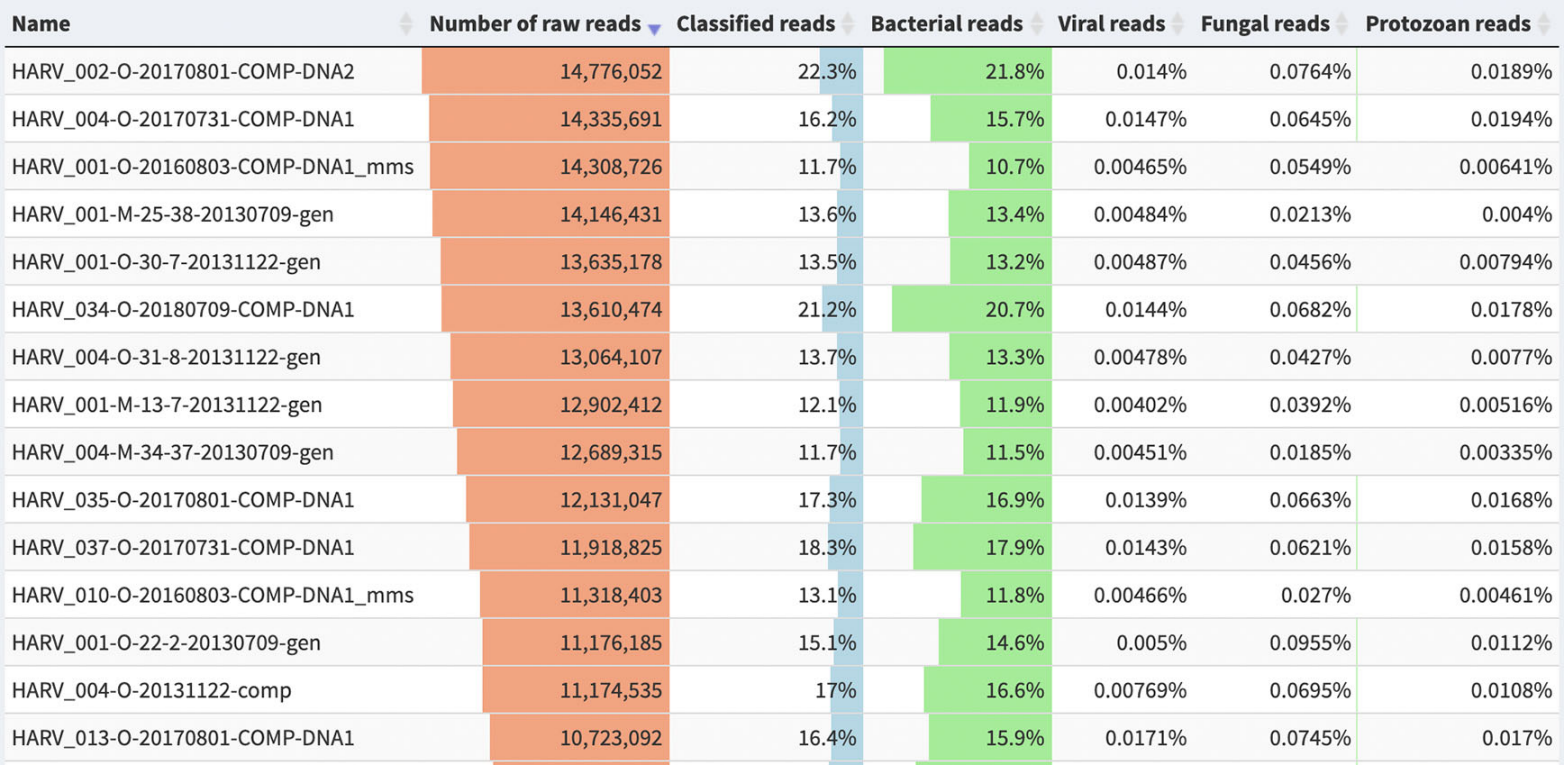
Percentage of metagenomic short reads assigned to high-level taxonomic categories. Samples are from the Harvard Forest site of the National Ecological Observatory Network (NEON). Reads were assigned using the *PlusPF* database (release 5/17/21), which includes sequences from archaea, bacteria, viral, plasmid, human, UniVec_Core, protozoa & fungi. Image generated using the visualization software Pavian ( Breitwieser & Salzberg, 2020).

The vast majority of soil sequences match to no known organism ( Figure 2). However, novel genomes can be assembled from metagenomes. These Metagenome-Assembled Genomes (MAGs) are more commonly assembled from human-associated samples, but they are quickly becoming a valuable resource for soil genomics: a recent collection of about 200 soil MAGs doubled the percentage of identifiable soil sequences, from 5% to 10% ( Nayfach *et al.*, 2020). See Chen *et al.* (2020) for an overview of the strengths and pitfalls of MAG assembly and publication.

**Figure 3.**
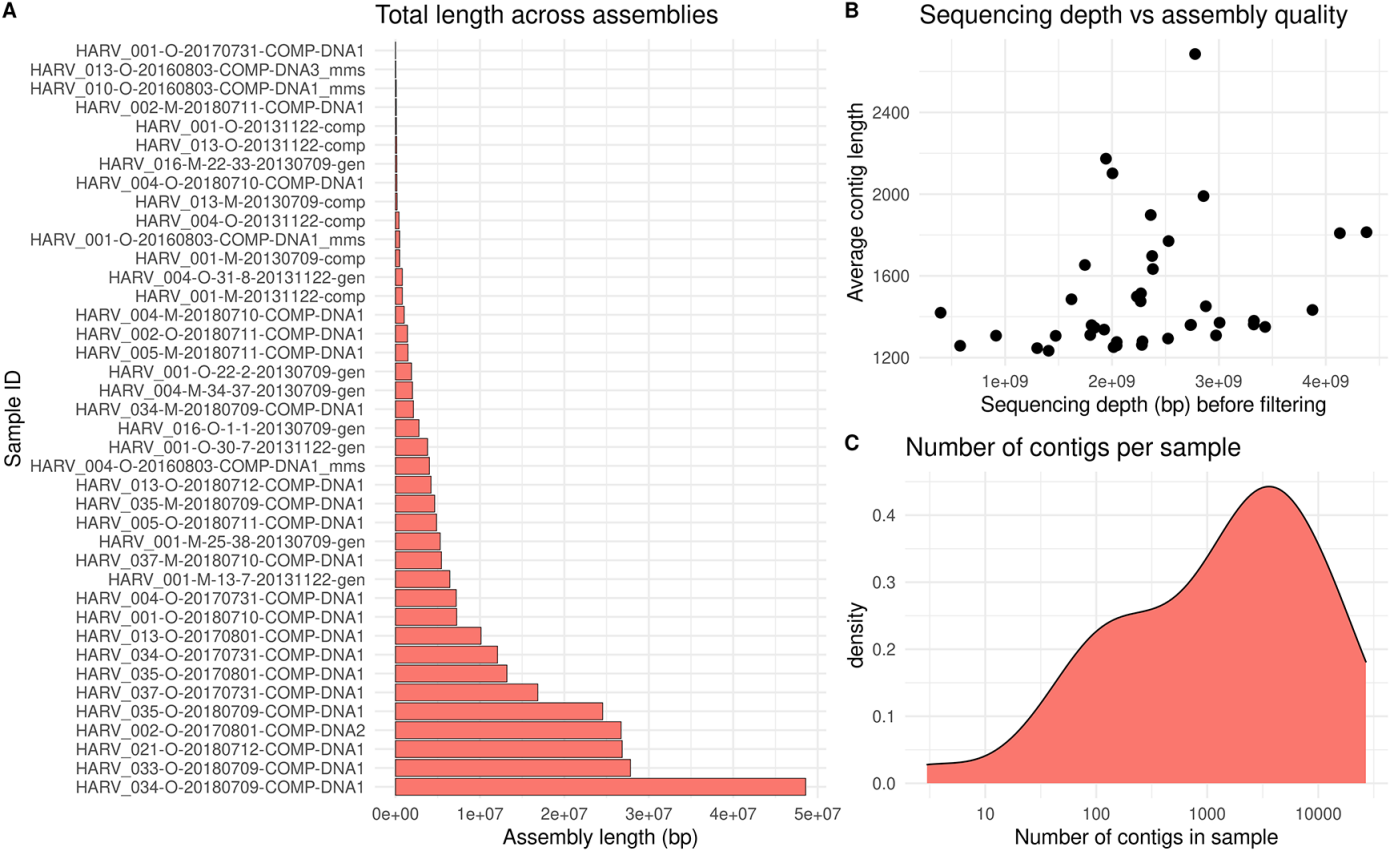
Results of contig assembly of short-read quality-filtered metagenomic samples. Contigs were assembled using the Megahit software, with samples from the Harvard Forest site of the National Ecological Observatory Network (NEON). The “meta-large” preset was used with a minimum contig length of 1000 base pairs (bp). a) Assembly length per sample, calculated as the sum of contig lengths within sample. b) Average contig length per sample, plotted against the sequencing depth before filtering. c) Density plot showing the number of contigs per sample.

Because MAGs are assembled directly from contigs, rather than grown in an experimental setting, they often have no cultured relatives, representing a hidden source of genetic diversity in the microbiome ( Nayfach *et al.*, 2020). For each putative genome, or “bin,” summary statistics are produced that estimate the completeness and possible contamination of the genome, using a set of genes that are expected to be “single-copy” within a genome ( Sieber *et al.*, 2018). Bins can be further refined manually, and genomes that are mostly complete with minimal contamination may be good candidates for submission to public databases ( Bowers *et al.*, 2017). High-quality MAGs can uncover entirely new lineages in the microbial tree of life ( Nayfach *et al.*, 2020).

Binning pipelines generally use a variety of separate binning tools, then refine and synthesize the best outputs from each tool. Bin refinement is essential for retrieving high-quality bins from soil than from other ecosystems, reflecting the challenges associated with soil bioinformatics ( Sieber *et al.*, 2018; Uritskiy *et al.*, 2018).

### 6.2 Considerations for NEON data

Many of the genomes in reference databases such as RefSeq and Genbank are actually chimeric (consisting of multiple organisms). Chimeric genomes are especially prevalent in metagenome-assembled genomes, with chimerism identified in up to 30% of “high-quality” MAGs. Differential coverage data (obtained from multiple samples) can very quickly identify chimeric organisms. This makes the extensive NEON dataset particularly valuable for identifying novel soil genomes. Chimeric genomes can be identified by visualizing genomes in Anvi’o, or by running tools such as GUNC ( Orakov *et al.*, 2021) that identify inconsistencies in the lineages of various genes.

### 6.3 Implementation

**Figure 4.**
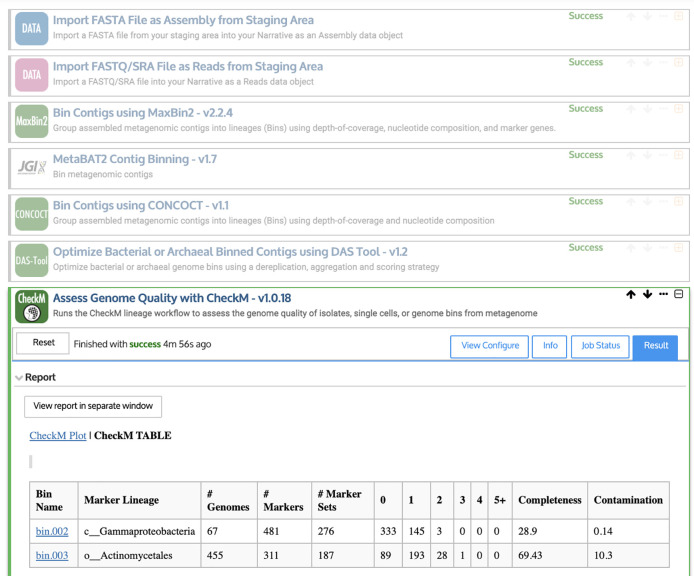
Example workflow for creating and evaluating Metagenome-Assembled Genomes (MAGs) using the KBase Narrative interface ( Arkin *et al.*, 2018). First, quality-controlled sequencing reads and assembled contigs are imported using upload modules. Then, contigs are binned into putative genomes (or “bins”) using MaxBin2 ( Wu *et al.*, 2016), MetaBAT2 ( Kang *et al.*, 2019), and CONCOCT ( Alneberg *et al.*, 2014). DAS Tool ( Sieber *et al.*, 2018) is used to identify the highest-quality bins. Finally, CheckM ( Parks *et al.*, 2015) reports the completeness and contamination (among other statistics) for each putative genome.

Genome binning is a well-supported feature of the KBase Predictive Biology platform, which was developed for microbiome analysis by the U.S. Department of Energy ( Arkin *et al.*, 2018). KBase links hundreds of different software tools using an online interface, which allows users to create “Narratives” for specific data analysis projects. In an example Narrative ( Figure 4), we combine the output from three tools, MaxBin2 ( Wu *et al.*, 2016), MetaBAT2 ( Kang *et al.*, 2019), and CONCOCT ( Alneberg *et al.*, 2014). As inputs, we use the contigs assembled by MEGAHIT, as well as the quality-controlled sequencing reads. DAS Tool ( Sieber *et al.*, 2018) and CheckM ( Parks *et al.*, 2015) report on genome quality. However, there is currently a limited number of supported software tools within KBase, so the next section presents a Snakemake-based approach for carrying out similar tasks.

### 6.4 Genome binning

Assembled contigs can be grouped into bins using information such as read overlap and differential abundance across samples. The following metaGEM rule calculates differential abundance, and feeds this information into three binning tools: CONCOCT, metaBAT, and MaxBin:

bash metaGEM.sh --task binning --local --cores 28


### 6.5 Bin evaluation & refinement

To determine genome completeness, the metaGEM pipeline evaluates bins using a reference database called CheckM. The compressed database file can be downloaded as part of the env_setup.sh script (see Implementation section). Once the “checkM” folder is in your metaGEM directory, decompress it by running:

mkdir checkM
tar -xvzf checkm_data_2015_01_16.tar.gz -C checkM
checkm data setRoot checkM # may take a moment to complete


Next, the outputs from Concoct, metaBAT, and MaxBin are refined by metaWrap. **The default cutoffs for keeping a genome are 50% minimum completeness and 10% maximum contamination. These values can be modified within the configuration file.** To run the bin refinement step:

bash metaGEM.sh --task binEvaluation --local


To view the resulting bin quality for each sample, go to the sample name within the “reassembled_bins” directory and inspect the generated plots.

### 6.6 Genome taxonomy

The newly-assembled genomes can be evaluated against genome databases to determine taxonomy. First, users must set up the Genome Taxonomy Database (GTDB) ( Parks *et al.*, 2020) and specify its location using the “GTDBTK_DATA_PATH” environment variable. For details on the download and installation of this database, see the GTDB-tk documentation ( Chaumeil *et al.*, 2020).

Once the database is setup, run the following command for taxonomic assignment:

bash metaGEM.sh --task gtdbtk --local


### 6.7 Additional analysis

Additional analysis - such as metabolic modeling, and simulating interactions between MAGs - can be carried out with metaGEM, but has more complex software requirements. Details on implementation are in the metaGEM readme.

## 7. Applications

The NEON microbial sampling structure was designed to allow researchers to connect microbial community structure and functional potential ( Stanish & Parnell, 2018). Complementary data streams can also be leveraged to link soil microbial data to ecosystem-level biogeochemical fluxes, plant growth, soil quality ( Vestergaard *et al.*, 2017) and more. We recommend Qin *et al.* (2021) for a discussion of the high-level questions that may be tackled using NEON soil microbial data; below we highlight a few topics and recommended resources.

### 7.1 Microbial community structure

NEON microbial data is well-suited for elucidating basic patterns in soil microbial ecology, such as the variation between communities at different spatial and temporal scales ( Qin *et al.*, 2021). The nested sampling, in which soil samples come from plots within each site, can be used to investigate spatial variability and autocorrelation among genes or taxa ( Averill *et al.*, 2021). Longer-term change in microbial communities could be studied by integrating multi-decadal data from the Long-Term Ecological Research ( LTER) program.

Shotgun metagenomes, which provide a snapshot of the entire genomic potential of a community, can be contrasted with amplicon sequencing, in which specific gene regions are amplified with the goal of distinguishing between taxa. NEON performs amplicon sequencing (NEON.DP1.10108.001) for soil fungi and bacteria, approximately 3 times per year at each site. These amplicon sequencing data can be accessed through the specialized neonMicrobe R package ( Qin *et al.*, 2021). To link amplicon sequences with metagenome-assembled genomes (MAGs; Section 6), MAGs must include the gene regions used for amplicon sequencing. Tools such as phyloFlash ( Gruber-Vodicka *et al.*, 2020) can be used to specifically assemble these gene regions and insert them into MAGs. This method provides an avenue for exploring the hidden diversity of the soil microbiome via genome assembly, while retaining the phylogenetic context of new genomes.

### 7.2 Biogeochemistry

The biogeochemical functions of soil microbes are poorly understood, despite their importance to global nutrient recycling. NEON measures many aspects of soil chemistry, which represents the nutrients available to microbial and plant communities. One-time characterizations of soil texture, bulk density, and detailed chemistry (including micronutrients such as zinc, iron, copper, etc.) are collected during the setup of each site (NEON.DP1.00096.001). Soil carbon and nitrogen are measured multiple times per year. (NEON.DP1.10086.001). Both datasets can be accessed using the neonUtilities R package or the NEON Data Portal. These can be used to investigate how microbial communities vary with chemical properties.

A subset of NEON metagenomes have an associated data stream on soil nitrogen transformations (NEON.DP1.10086.001), usually measured at each site once every five years. To calculate microbial rates of nitrogen mineralization and nitrification, soils are incubated for a month. Initial and final pools of ammonium, nitrites, and nitrates can be converted into daily transformation rates using the neonNTrans R package ( Weintraub, 2021). To link these nitrogen transformation rates to microbial data, users can estimate the abundances of pathway genes from NCycDB (Section 5.3), and match datasets with the dnaSampleID sample identifier. Genes that encode for enzymes like ammonia monooxygenase (AMO) are often used as proxies for nitrogen transformation activity, though the relationships between gene presence and functional activity are poorly characterized ( Rocca *et al.*, 2015). NEON's soil nitrogen and microbial data can be used to clarify the strength of gene-function relationships across diverse biomes.

### 7.3 Plant communities

The soil microbiome is intimately linked with plant communities, which rely on (or compete with) soil microbes for nutrients (Bo *et al.*, 2022). NEON soil microbial data is collected alongside detailed inventories of plant species (DP1.10058.001), phenology (DP1.10055.001), tree biomass (DP1.10098.001), root biomass (DP1.10066.001), and root stable isotopes (DP1.10099.001). Summaries of plant diversity metrics at multiple spatial resolutions are available using the neonDiversity R package (Mahood, 2020). These data streams could be used to answer long-standing questions about spatio-temporal associations between plants and microbes ( O'Brien *et al.*, 2021). For instance, soils form the “seed bank” from which plants recruit microbial symbionts (Bo *et al.*, 2022). The metabolic capacity of these symbionts can change the growth and stress tolerance of plants ( Ravanbakhsh *et al.*, 2019). Soil metagenomes could be used to identify key microbial genes or symbionts affecting plant distributions across ecosystems ( Cregger *et al.*, 2021).

### 7.4 Bioinformatics

Major challenges in soil bioinformatics include the lack of reference databases and specialized analysis tools, with different pipelines often leading to divergent conclusions ( Pauvert *et al.*, 2019). NEON sequences can be used to develop bioinformatics pipelines that work well across biologically and physically heterogeneous soil biomes. Currently available pipelines that work well on some soils may perform poorly on other soils, because soil chemistry affects sequencing library preparation and can lead to downstream biases in sequence data. For instance, guanine-cytosine (GC) content of genomic regions can add bias to sample preparation steps, such as DNA lysing and sequencing ( Benjamini & Speed, 2011). GC content is related, however, to temperature and nutrient conditions, and varies between species. While many bioinformatic tools attempt to correct for GC bias, these normalization steps may not be equally important for different soils. By freely providing sequences from a variety of biomes, researchers can calibrate tools against a reference dataset that reflects the full diversity of soils. More generally, NEON shotgun metagenomes can be used to investigate how variation in bioinformatic pipeline decisions affect ecological inferences. They may also act as a valuable resource for soil bioprospecting efforts, which use bioinformatic approaches to identify bioactive compounds with potential medical or industrial value ( Vuong *et al.*, 2022).

## Data availability

Raw metagenomics sequencing data is published in RELEASE-2021 as DP1.10107.001 from the National Ecological Observatory Network ( https://data.neonscience.org/data-products/explore). All other data is previously published and cited throughout the paper.

## Software availability

Bioconductor packages available at https://www.bioconductor.org/. CRAN packages available at https://cran.r-project.org/. metaGEM software is available at https://github.com/franciscozorrilla/metaGEM and the version used for this publication is archived at https://doi.org/10.5281/zenodo.4707723.

## Author contributions

ZRW, BH, and JLN developed the software tools and tested the workflow. ZRW and BH wrote the initial manuscript draft; all authors contributed to revisions. JMB and MD provided project supervision and obtained funding.
