# Supplementary material for: The National Ecological Observatory Network’s soil metagenomes: assembly and basic analysis
Source: F1000Res. 2022 Mar 23;10:299. Originally published 2021 Apr 19. [Version 2] doi: 10.12688/f1000research.51494.2 (PMC9178279; doi:10.12688/f1000research.51494.2)
Supplement: Peer review history

# Peer review: Reviewer response for version 2

**DOI:** 10.5256/f1000research.79544.r139218

**Published:** 2022-06-08

**Authors:** Olubukola Oluranti Babalola

**Affiliations:** 1 Food Security and Safety Focus Area, Faculty of Natural and Agricultural Science, North-West University, Mmabatho, South Africa

The manuscript presents analysis and workflows of the NEON metagenomics data collected annually and how the datasets can be interrogated. The manuscript reported on the software that offers users, who are not yet confident enough, to build their pipeline from the start to use the software to analyze metagenomics, especially shotgun datasets. The software information has been improved to give room for the reproducibility of data. Each step involved in implementing the software was adequately described to ensure replication of the output.

 A little addition would have been to present a user-friendly interface for beginners, who may not be familiar with or confident in using command lines, just as the part of KBase that was part of the workflow. Future studies can look into that.

Is the rationale for developing the new method (or application) clearly explained?

Yes

Is the description of the method technically sound?

Yes

Are the conclusions about the method and its performance adequately supported by the findings presented in the article?

Partly

If any results are presented, are all the source data underlying the results available to ensure full reproducibility?

Yes

Are sufficient details provided to allow replication of the method development and its use by others?

Yes

Reviewer Expertise:

Plant/Soil Microbe Interactions

I confirm that I have read this submission and believe that I have an appropriate level of expertise to confirm that it is of an acceptable scientific standard.

# Peer review: Reviewer response for version 1

**DOI:** 10.5256/f1000research.54670.r84561

**Published:** 2021-07-19

**Authors:** William Nelson

**Affiliations:** 1 Biological Sciences Division, Pacific Northwest National Laboratory, Richland, WA, USA

**Rationale:**

 My main question is who is the audience for this pipeline? Is this intended to be used by students to learn some metagenomic analysis and how the NEON data set can be interrogated? Or is this intended to be used by researchers, in which case I think the downstream annotation and analysis components are somewhat thin. Is this officially recognized by NEON as a standard pipeline that will enable comparison between analyses? I don't wish to sound dismissive, but this reads like a Yet-Another-Metagenomics-Pipeline paper, which on one hand is fine - there's nothing technically or scientifically wrong with it - but this would be a more impactful report if the purpose behind it was more strongly presented.

**Description:**

 There is nothing wrong with the description of the various steps, but the descriptions are superficial. There is little discussion of why the methods were chosen and what their strengths and weaknesses are.

**Replication:**

 The code blocks are great, but the formatting rendered incorrectly in my browser (Firefox) - newlines were not present, making it hard to interpret what the actual commands are. Also, I tried to follow along with those commands on our institutional computing cluster and got stuck on the installation of sunbeam. I was able to install sunbeam on my desktop server, but the test of the install failed. I went ahead and tried to follow the analysis anyway, but ran into multiple problems. Just a caveat that providing the commands doesn't ensure replicability. 

**A few other comments:**

 End of Dataset description: "TOS Science Design for Terrestrial Microbial Diversity, NEON.DOC.000908" - What is this?

 The comment about miniconda, "this command may work", is likely to be confusing. Might be best just to say that anaconda is required and to talk to local IT about its availability and how to use it.

 The transition between section 1.2 and 2 should make it clearer that section 1.2 was describing constructing the configuration file and sections 2 through 5 are describing the individual steps that make up the sunbeam pipeline. As it reads now, it could be interpreted that the QC step is subsequent to the sunbeam run.

 Is section 4.1b missing a code block?

 I did not understand what you meant by "We use the homolog protein genes to construct our reference database." in section 5.

 The Bowers 2017 reference appears to be missing from the bibliography.

Is the rationale for developing the new method (or application) clearly explained?

Partly

Is the description of the method technically sound?

Partly

Are the conclusions about the method and its performance adequately supported by the findings presented in the article?

Yes

If any results are presented, are all the source data underlying the results available to ensure full reproducibility?

Yes

Are sufficient details provided to allow replication of the method development and its use by others?

Partly

Reviewer Expertise:

I have 20 years experience performing microbial genomic and metagenomic analysis, including assembly, binning and annotation.

I confirm that I have read this submission and believe that I have an appropriate level of expertise to confirm that it is of an acceptable scientific standard, however I have significant reservations, as outlined above.

# Response

**Published:** 2021-11-22

**Authors:** Zoey Werbin

**Affiliations:** Boston University, USA

- *My main question is who is the audience for this pipeline? Is this intended to be used by students to learn some metagenomic analysis and how the NEON data set can be interrogated? Or is this intended to be used by researchers, in which case I think the downstream annotation and analysis components are somewhat thin. Is this officially recognized by NEON as a standard pipeline that will enable comparison between analyses? I don't wish to sound dismissive, but this reads like a Yet-Another-Metagenomics-Pipeline paper, which on one hand is fine - there's nothing technically or scientifically wrong with it - but this would be a more impactful report if the purpose behind it was more strongly presented.*

Thank you for identifying these deficiencies within the manuscript. Our intended audience is both students and researchers working with NEON soil metagenomes. We have stated this explicitly in the last paragraph of the Introduction to the article, and strengthened each section of the paper to increase its value to these groups. Specifically, we have added subsections titled "Background and Rationale" and "Considerations for NEON data" to each analysis section. We plan to submit this revised manuscript for inclusion as a NEON community resource. 

- *There is nothing wrong with the description of the various steps, but the descriptions are superficial. There is little discussion of why the methods were chosen and what their strengths and weaknesses are.*

Each step has now been supplemented with descriptions of our preferred methods as well as the strengths and weaknesses of alternative methods (in "Background and Rationale"). We describe which methods have or have not been benchmarked or optimized for soil metagenomes, specifically, as well as their usefulness for the NEON dataset, given the properties of the data (in "Considerations for NEON data").

- *The code blocks are great, but the formatting rendered incorrectly in my browser (Firefox) - newlines were not present, making it hard to interpret what the actual commands are. Also, I tried to follow along with those commands on our institutional computing cluster and got stuck on the installation of sunbeam. I was able to install sunbeam on my desktop server, but the test of the install failed. I went ahead and tried to follow the analysis anyway, but ran into multiple problems. Just a caveat that providing the commands doesn't ensure replicability. *

Great points. In response to this and to the comments of Reviewer #1, we have adjusted our specific bioinformatic methods to address Sunbeam installation issues. We now recommend the stable branch of the metaGEM pipeline, which has run successfully in multiple Linux environments. The code blocks have all been shortened to improve readability and cross-browser formatting.

- *End of Dataset description: "TOS Science Design for Terrestrial Microbial Diversity, NEON.DOC.000908" - What is this?*

The citation for this sampling protocol document has been changed to "Stanish & Parnell, 2018", with the full protocol version information within the Works Cited.

- *The comment about miniconda, "this command may work", is likely to be confusing. Might be best just to say that anaconda is required and to talk to local IT about its availability and how to use it.*

The sentence on miniconda requirements has been revised to point readers to their system administrators.

- *The transition between section 1.2 and 2 should make it clearer that section 1.2 was describing constructing the configuration file and sections 2 through 5 are describing the individual steps that make up the sunbeam pipeline. As it reads now, it could be interpreted that the QC step is subsequent to the sunbeam run.*

This recommendation is no longer relevant, given our shift in methods and manuscript organization.

- *Is section 4.1b missing a code block?*

This section is no longer present, given our shift in methods and manuscript organization.

- *I did not understand what you meant by "We use the homolog protein genes to construct our reference database." in section 5.*

This section is no longer present, given our shift in methods and manuscript organization.

- *The Bowers 2017 reference appears to be missing from the bibliography.*

This reference has been added to the bibliography.

# Peer review: Reviewer response for version 1

**DOI:** 10.5256/f1000research.54670.r83581

**Published:** 2021-06-28

**Authors:** Naupaka Zimmerman

**Affiliations:** 1 Department of Biology, University of San Francisco, San Francisco, CA, USA

This is a timely and valuable contribution that has the potential to aid in the use of NEON data by a wider audience. The core approach (using Sunbeam, a snakemake pipeline, to analyze NEON metagenomics data) seems like a good one, and will offer advantages to users who are not yet comfortable enough to develop their own such pipeline from scratch.

 While in general the approach is a good one and the need for the tool is real and well-articulated by the authors, there are a number of aspects that could be improved to maximize the value of this contribution. I will outline a few here, but I was unable to complete the full pipeline in my testing using the example data specified in the manuscript, and so I am not able to comment on all aspects of the pipeline at this time. I would be happy to do another view and assessment after hearing from the authors.

 I outline some suggestions below:

 In the last paragraph of the introduction, I would encourage the authors to revise this sentence: "The pipeline that we present here is designed to complement existing NEON educational resources, such that users without prior bioinformatics experience may use this dataset to learn about microbial communities within the soil." The background skills that are necessary to successfully understand and implement the approach outlined here is not trivial and I don't think it's exactly best suited for someone "without prior bioinformatics experience". I think such a user would more likely need a graphical interface that did not presume comfort with the *nix command line etc. I think the approach outlined here is a valuable contribution because it targets users who may have some comfort with programmatic and command-line approaches, but does not yet have the skill to develop a flexible pipeline themselves.

 In the methods section, first paragraph, I think I would revise to be more careful with tenses. In some cases the collection protocols will remain mostly unchanged (e.g. I don't think NEON is planning to add any core sites), but other things may change (the kits that they use, the sequencing depth or sequencer used, etc. Since NEON is a 30 year project, it might help the manuscript's longevity if this paragraph were worded to reflect possible future methodological changes.

 I might encourage a mention or a suggestion that users use tmux or screen to run pipelines like this is they are connected to a remote server over something like ssh. If the connection drops during a many hours long pipeline, it can be quite frustrating.

 In step 1.2, why do you suggest the use of the develop branch of Sunbeam? Isn't that more likely to include breaking changes that will be overly challenging for the target audience? Perhaps this could be adjusted to use a stable branch or version, and the text could highlight the develop branch alternative for those willing to trade troubleshooting time in exchange for quicker access to more advanced features.

 For downloading the config file, it might be better to pull from an archival version of the file instead of the github version, or at the least include a version at a specific commit and not just the main branch, so that it remains stable. Otherwise either the code could break, or the authors would need to continually update the configuration to track with software changes.

 In my testing of the approach in the manuscript, I am unable to get past the tests that occur after the installation of Sunbeam (`bash tests/run_tests.bash`). The tests repeatedly fail with segmentation faults during either the megahit or kraken steps. This is on an Ubuntu 20.04 machine with lots of RAM/disk space/cores. I am not sure where the issue is, and I would consider myself reasonably able to troubleshoot such problems, so I am concerned that similar problems might arise and be too challenging for the target audience/user. I would be happy to work with the authors in more detail to resolve this problem (share log files, etc). I shall share them via a comment when I am able to.

 Overall, I think this is a valuable contribution that fills a need in the community and uses a good approach to do so. However, in its current form, I cannot successfully run the example code, even on the recommended sample files, and so I have concerns with the brittleness of the approach outlined. I'd encourage the authors to do some additional testing on other machines and settings, and/or build some more resilience into the installation walkthrough so that the average target user is able to make use of this contribution.

Is the rationale for developing the new method (or application) clearly explained?

Yes

Is the description of the method technically sound?

Partly

Are the conclusions about the method and its performance adequately supported by the findings presented in the article?

Partly

If any results are presented, are all the source data underlying the results available to ensure full reproducibility?

Yes

Are sufficient details provided to allow replication of the method development and its use by others?

Partly

Reviewer Expertise:

Environmental microbial ecology, including specific experience in bioinformatics and pipelines, and several years of experience working with large NEON sequencing datasets.

I confirm that I have read this submission and believe that I have an appropriate level of expertise to confirm that it is of an acceptable scientific standard, however I have significant reservations, as outlined above.

# Response

**Published:** 2021-11-22

**Authors:** Zoey Werbin

**Affiliations:** Boston University, USA

Original reviewer comments are italicized. 

- *This is a timely and valuable contribution that has the potential to aid in the use of NEON data by a wider audience. The core approach (using Sunbeam, a snakemake pipeline, to analyze NEON metagenomics data) seems like a good one, and will offer advantages to users who are not yet comfortable enough to develop their own such pipeline from scratch. While in general the approach is a good one and the need for the tool is real and well-articulated by the authors, there are a number of aspects that could be improved to maximize the value of this contribution. I will outline a few here, but I was unable to complete the full pipeline in my testing using the example data specified in the manuscript, and so I am not able to comment on all aspects of the pipeline at this time. I would be happy to do another view and assessment after hearing from the authors.*
  Thank you for highlighting the issues with the reproducibility of the pipeline we outlined. Due to the referenced issues with installing software, we have switched to a similar Snakemake pipeline (metaGEM) that has been tested on various computing systems. We describe this new pipeline in the "Implementation" section of the revised manuscript.
- In the last paragraph of the introduction, I would encourage the authors to revise this sentence: "The pipeline that we present here is designed to complement existing NEON educational resources, such that users without prior bioinformatics experience may use this dataset to learn about microbial communities within the soil." The background skills that are necessary to successfully understand and implement the approach outlined here is not trivial and I don't think it's exactly best suited for someone "without prior bioinformatics experience". I think such a user would more likely need a graphical interface that did not presume comfort with the *nix command line etc. I think the approach outlined here is a valuable contribution because it targets users who may have some comfort with programmatic and command-line approaches, but does not yet have the skill to develop a flexible pipeline themselves.
  This sentence has been revised to reflect that our audience is those with basic bioinformatics experience. Further, each section of the manuscript has been expanded to include a thorough description of the rationale for various decisions in the subsections "Background and Rationale" and "Considerations for NEON data", so that this can be a more useful introductory guide to soil metagenomics. 
- *In the methods section, first paragraph, I think I would revise to be more careful with tenses. In some cases the collection protocols will remain mostly unchanged (e.g. I don't think NEON is planning to add any core sites), but other things may change (the kits that they use, the sequencing depth or sequencer used, etc. Since NEON is a 30 year project, it might help the manuscript's longevity if this paragraph were worded to reflect possible future methodological changes.*
  Tenses in the "Dataset description" section have been modified to reflect that the reported sampling and sequencing protocols are accurate as of 2021. We state that this bioinformatics protocol is intended for short-read data specifically, and that NEON protocols may shift in the future.
- *I might encourage a mention or a suggestion that users use tmux or screen to run pipelines like this is they are connected to a remote server over something like ssh. If the connection drops during a many hours long pipeline, it can be quite frustrating.*
  We now reference tmux and screen in Implementation, within the sub-section "Local vs cluster analysis".
- *In step 1.2, why do you suggest the use of the develop branch of Sunbeam? Isn't that more likely to include breaking changes that will be overly challenging for the target audience? Perhaps this could be adjusted to use a stable branch or version, and the text could highlight the develop branch alternative for those willing to trade troubleshooting time in exchange for quicker access to more advanced features.*
  Due to our shift in methods, we no longer use either the develop or stable branch of Sunbeam. At the time of writing, however, the develop branch had implemented a potential fix for the segmentation fault errors, but it did not resolve errors on all operating systems. We hope the local and cluster options for running the metaGEM pipeline will also help with reducing troubleshooting time.
- *For downloading the config file, it might be better to pull from an archival version of the file instead of the github version, or at the least include a version at a specific commit and not just the main branch, so that it remains stable. Otherwise either the code could break, or the authors would need to continually update the configuration to track with software changes.*
  With our shift from Sunbeam to metaGEM, we decided to remove the example configuration file. The configuration file that comes installed with metaGEM primarily needs file paths to be modified by the user, whereas most parameters can be left as-is. Throughout the text, we've bolded sentences that instruct the user to modify the configuration filepaths.

- *In my testing of the approach in the manuscript, I am unable to get past the tests that occur after the installation of Sunbeam (`bash tests/run_tests.bash`). The tests repeatedly fail with segmentation faults during either the megahit or kraken steps. This is on an Ubuntu 20.04 machine with lots of RAM/disk space/cores. I am not sure where the issue is, and I would consider myself reasonably able to troubleshoot such problems, so I am concerned that similar problems might arise and be too challenging for the target audience/user. I would be happy to work with the authors in more detail to resolve this problem (share log files, etc). I shall share them via a comment when I am able to.*

*Overall, I think this is a valuable contribution that fills a need in the community and uses a good approach to do so. However, in its current form, I cannot successfully run the example code, even on the recommended sample files, and so I have concerns with the brittleness of the approach outlined. I'd encourage the authors to do some additional testing on other machines and settings, and/or build some more resilience into the installation walkthrough so that the average target user is able to make use of this contribution.*

 These are excellent points and led to a dramatic shift in the focus and implementation of this analysis pipeline. The main text of the manuscript now focuses on the various options available to users for each step of soil metagenomic analysis, and describes issues specific to soil ecology and the NEON dataset specifically. The code at the end of each section is now an example of how these decisions may be implemented via specific tools. For this revision, we have communicated with the developers of the tools mentioned (metaGEM and Toolchest) and are confident that these tools will maintain resilience in the coming years. We hope this sufficiently addresses problems of brittleness.
